# Supplementary material for: NATPS: Nonadiabatic Transition Path Sampling Using the Time-Reversible Mapping Approach to Surface Hopping
Source: J Phys Chem Lett. 2026 May 13;17(21):6014–23. doi: 10.1021/acs.jpclett.6c00910 (PMC13224161; doi:10.1021/acs.jpclett.6c00910)
Supplement: Supplementary file 1 [file jz6c00910_si_001.pdf]

Supporting Information: "NATPS: Nonadiabatic Transition Path Sampling Using the Time-Reversible Mapping Approach to Surface Hopping"

Xiran Yang,[†,‡] Madlen Maria Reiner,[¶,§,‡] Brigitta Bachmair,[†,‡] Leticia González,[*,†,‡] Johannes C. B. Dietschreit,[*,†,‡] and Christoph Dellago[*,¶,‡]

†*Institute of Theoretical Chemistry, Faculty of Chemistry, University of Vienna, Währinger Straße 17, 1090 Vienna, Austria*

‡*Research Platform on Accelerating Photoreaction Discovery (ViRAPID), University of Vienna, Währinger Straße 17, 1090 Vienna, Austria*

¶*Faculty of Physics, University of Vienna, 1090 Vienna, Austria*

§*Vienna Doctoral School in Physics, University of Vienna, Boltzmanngasse 5, 1090 Vienna, Austria*

E-mail: leticia.gonzalez@univie.ac.at; johannes.dietschreit@univie.ac.at; christoph.dellago@univie.ac.at

Contents

S1 Invariant Distribution of MASH **S-4**

S1.1 Equations of motion in phase space S-5

S1.2 Equations of motion for the spin vector S-6

S1.3 Stationary Distribution S-7

S1.4 Fokker–Planck equation for $(\mathbf{q}, \mathbf{p}, \vec{S})$ S-10

S1.4.1 Case “away from the equator” ($S_z \neq 0$) S-15

S1.4.2 Case “at the equator” ($S_z = 0$) S-16

S2 Phase space volume conservation (for $\gamma = 0$) **S-18**

S3 Inverting MASH Dynamics **S-20**

S3.1 MASH equations of motion S-20

S3.2 Interpretation of time reversal S-20

S3.3 Summary of Time Inversion Transformations S-21

S4 Detailed Balance **S-22**

S4.1 Time-reversal parity operator S-23

S4.2 Conditions for Detailed Balance S-23

S4.3 Check condition Eq. (S80) S-24

S4.3.1 Case 1: positions ($i = 1, \ldots, N$, $\varepsilon_i = 1$) S-24

S4.3.2 Case 2: momenta ($i = N+1, \ldots, 2N$, $\varepsilon_i = -1$) S-24

S4.3.3 Case 3: Spin components ($i = 2N+1$, $2N+2$, $2N+3$) S-25

S4.4 Check condition Eq. (S81) S-26

S5 Relation of the dynamics of $\vec{S}$ to that of c_+ and c_- **S-28**

S6 Reflection of $\vec{S}$ at frustrated transition **S-31**

S7 Inversion of Coefficients **S-33**

S7.1 Proof S-34

S8 Local Diabatization **S-36**

S9 Numerical Accuracy with Naive Velocity-Verlet **S-37**

S10 The Uhlenbeck-Ornstein perturbation and detailed balance **S-39**

S11 Analytical Properties of the Model System **S-41**

S11.1Adiabatic Energies S-41

S11.2Nonadiabatic Coupling S-41

S11.3Dependence on the Electronic Coupling S-42

S12 Proof of Rapid Equilibration **S-43**

S13 The generation of lineage plot with TPS **S-44**

References **S-46**

S1 Invariant Distribution of MASH

To find the stationary distribution generated by the Mapping Approach to Surface Hopping (MASH), we consider a two-state system with the energy function E,

$$E(\mathbf{q}, \mathbf{p}, \vec{S}) = \sum_{i=1}^{N} \frac{p_i^2}{2m_i} + \bar{V}(\mathbf{q}) + V_z(\mathbf{q}) \, \mathrm{sign}(S_z) \tag{S1}$$

with $2N + 3$ degrees of freedom, representing the positions $\mathbf{q} = (q_1, q_2, \ldots, q_N)^\top$, conjugated momenta $\mathbf{p} = (p_1, p_2, \ldots, p_N)^\top$, masses m_i, and the spin vector $\vec{S}$,

$$\vec{S} = \begin{pmatrix} S_x \\ S_y \\ S_z \end{pmatrix}$$

The sign of S_z indicates whether the spin vector $\vec{S}$ is in the northern (+) or southern (−) hemisphere of the Bloch sphere. The hemispheres correspond to the upper and lower potential energy surfaces (PESs), V_+ and V_-, which are two different electronic states of the system, e.g. ground and excited state.

The state–independent potential $\bar{V}(\mathbf{q})$ is taken to be the average of the two states

$$\bar{V}(\mathbf{q}) = \frac{V_-(\mathbf{q}) + V_+(\mathbf{q})}{2} \tag{S2}$$

and the difference-potential $V_z(\mathbf{q})$ is

$$V_z(\mathbf{q}) = \frac{V_+(\mathbf{q}) - V_-(\mathbf{q})}{2} \tag{S3}$$

hence, the potential energy gap between upper and lower PESs corresponds to $2V_z(\mathbf{q})$. Using

Eq. (S2) and Eq. (S3) the ground and excited state potentials can be written as

$$V_-(\mathbf{q}) = \bar{V}(\mathbf{q}) - V_z(\mathbf{q}) \tag{S4}$$

$$V_+(\mathbf{q}) = \bar{V}(\mathbf{q}) + V_z(\mathbf{q}) \tag{S5}$$

S1.1 Equations of motion in phase space

The equations of motion in phase space (i.e., for positions q_i and conjugated momenta p_i) in the MASH approach are given as

$$\dot{q}_i = \frac{p_i}{m_i} \tag{S6}$$

$$\dot{p}_i = \bar{F}_i(\mathbf{q}) + F_i^{\mathrm{MASH}}(\mathbf{q}, \vec{S}) \tag{S7}$$

with mass m, where $\bar{F}$ is the average force induced by the state-independent potential $\bar{V}$,

$$\bar{F}_i(\mathbf{q}) = -\frac{\partial \bar{V}(\mathbf{q})}{\partial q_i} \tag{S8}$$

and F^{MASH} (cf. Eq. (8b) in[S1]) includes the derivative of the difference-potential V_z as well as a nonadiabatic coupling term

$$F_i^{\mathrm{MASH}}(\mathbf{q}, \vec{S}) = -\frac{\partial V_z(\mathbf{q})}{\partial q_i} \operatorname{sign}(S_z) + 4V_z(\mathbf{q})\, d_i(\mathbf{q})\, S_x\, \delta(S_z) \tag{S9}$$

with $d_i(\mathbf{q})$ being the nonadiabatic coupling vector. The delta function $\delta(S_z)$ activates the last term on the right hand side when the spin vector $\vec{S}$ crosses the equator of the Bloch sphere, i.e., the hemisphere changes from northern to southern or vice versa.

If the system is coupled to a heat bath, the equations of motion become

$$\dot{q}_i = \frac{p_i}{m_i} \tag{S10}$$

$$\dot{p}_i = -\frac{\partial \bar{V}(\mathbf{q})}{\partial q_i} - \frac{\partial V_z(\mathbf{q})}{\partial q_i}\,\mathrm{sign}(S_z) + 4V_z(\mathbf{q})\,d_i(\mathbf{q})S_x\,\delta(S_z) - \frac{\gamma}{m_i}p_i + \mathcal{F}_i(t) \tag{S11}$$

where a friction term with friction constant γ and random force $\mathcal{F}_i(t)$ are introduced. The random force fulfills the fluctuation dissipation theorem, such that

$$\langle \mathcal{F}_i(t)\,\mathcal{F}_j(t')\rangle = 2\gamma k_B T\,\delta_{ij}\,\delta(t-t') \tag{S12}$$

for times t and t' with Boltzmann constant k_B, where T is the absolute temperature of the bath.

S1.2 Equations of motion for the spin vector

The equations of motion for the spin vector $\vec{S}$ are

$$\dot{S}_x = 2\left(\sum_i d_i(\mathbf{q})\,\frac{p_i}{m_i}\right)S_z - 2V_z(\mathbf{q})\,S_y \tag{S13}$$

$$\dot{S}_y = 2V_z(\mathbf{q})\,S_x \tag{S14}$$

$$\dot{S}_z = -2\left(\sum_i d_i(\mathbf{q})\,\frac{p_i}{m_i}\right)S_x \tag{S15}$$

They can be re-written in vector form as

$$\dot{\vec{S}} = \vec{H}\times\vec{S}, \qquad \vec{H} = \begin{pmatrix} 0 \\ 2\sum_i d_i(\mathbf{q})\frac{p_i}{m_i} \\ 2V_z(\mathbf{q}) \end{pmatrix} \tag{S16}$$

with the cross product structure

$$\vec{H} \times \vec{S} = \begin{pmatrix} H_x \\ H_y \\ H_z \end{pmatrix} \times \begin{pmatrix} S_x \\ S_y \\ S_z \end{pmatrix} = \begin{pmatrix} H_y S_z - H_z S_y \\ H_z S_x - H_x S_z \\ H_x S_y - H_y S_x \end{pmatrix} \tag{S17}$$

Here $\vec{H}$ can be interpreted as an effective magnetic field. Since $\dot{\vec{S}} \perp \vec{S}$, the magnitude of $\vec{S}$ is conserved,

$$\frac{d}{dt}|\vec{S}| = 0$$

Notice that the heat bath does not couple to the virtual spin degrees of freedom. We can interpret the motion of the spin vector as a precession around the axis $\vec{H}$, where H_y describes the nonadiabatic coupling and the energy difference between ground and excited state enters via V_z (see Eq. (S3)).

S1.3 Stationary Distribution

We would like to prove that the stationary distribution of this dynamics is the distribution

$$\rho(\mathbf{q}, \mathbf{p}, \vec{S}) \propto \exp\left[-\beta\left(\sum_i \frac{p_i^2}{2m_i} + \bar{V}(\mathbf{q}) + V_z(\mathbf{q})\,\mathrm{sign}(S_z)\right)\right] \delta(|\vec{S}| - 1) \tag{S18}$$

with the first term stemming from the Boltzmann distribution and the delta function confining the virtual spin vector to the surface of the Bloch sphere. $\beta \equiv 1/k_B T$ is the inverse temperature.

The distribution on the Bloch sphere is

$$\begin{aligned}\rho(\vec{S}) &= \int \mathrm{d}\mathbf{q}\,\mathrm{d}\mathbf{p}\,\rho(\mathbf{q},\mathbf{p},\vec{S}) \\ &= \frac{1}{Q}\int \mathrm{d}\mathbf{p}\ \exp\left[-\beta\sum_i \frac{p_i^2}{2m_i}\right]\int \mathrm{d}\mathbf{q}\ \exp\left[-\beta\left(\bar{V}(\mathbf{q})+V_z(\mathbf{q})\mathrm{sign}(S_z)\right)\right]\ \delta(|\vec{S}|-1)\end{aligned} \tag{S19}$$

where Q is the partition function. We denote the configuration integral for the full two-state system as

$$\frac{1}{Z} = \frac{1}{Q}\int \mathrm{d}\mathbf{p}\ \exp\left[-\beta\sum_i \frac{p_i^2}{2m_i}\right] \tag{S20}$$

and introduce Z_α as the integral over only PES V_α with $\alpha \in \{+,-\}$,

$$Z_+ = \int \mathrm{d}\mathbf{q}\ \exp\left[-\beta\left(\bar{V}(\mathbf{q})+V_z(\mathbf{q})\right)\right] \tag{S21}$$

$$Z_- = \int \mathrm{d}\mathbf{q}\ \exp\left[-\beta\left(\bar{V}(\mathbf{q})-V_z(\mathbf{q})\right)\right] \tag{S22}$$

For the case $S_z > 0$, i.e., the spin vector on the northern (excited state) hemisphere, we get

$$\rho(\vec{S}) = \frac{1}{Z}\int \mathrm{d}\mathbf{q}\ \exp\left[-\beta\left(\bar{V}(\mathbf{q})+V_z(\mathbf{q})\right)\right]\ \delta(|\vec{S}|-1) = \frac{Z_+}{Z}\,\delta(|\vec{S}|-1) \tag{S23}$$

and accordingly, for the case $S_z < 0$, i.e., the spin vector on the southern (ground state) hemisphere,

$$\rho(\vec{S}) = \frac{1}{Z}\int \mathrm{d}\mathbf{q}\ \exp\left[-\beta\left(\bar{V}(\mathbf{q})-V_z(\mathbf{q})\right)\right]\ \delta(|\vec{S}|-1) = \frac{Z_-}{Z}\,\delta(|\vec{S}|-1) \tag{S24}$$

Eqs. (S23) and (S24) show that the probability density of $\vec{S}$ is uniform on each hemisphere. The relative population of the northern (excited state) and the southern (ground state) hemisphere is

$$\frac{P_+}{P_-} = \frac{Z_+}{Z_-} = \frac{\int \mathrm{d}\mathbf{q}\ e^{-\beta V_+(\mathbf{q})}}{\int \mathrm{d}\mathbf{q}\ e^{-\beta V_-(\mathbf{q})}} \tag{S25}$$

by using eqs. (S4) and (S5). Using the difference potential V_z (see Eq. (S3)), the relative population of the hemispheres is proportional to the exponentiated free energy difference ΔF between both states

$$\frac{P_+}{P_-} = e^{-\beta \Delta F} \ . \tag{S26}$$

We can rewrite the ratio Eq. (S25) as ensemble averages

$$\langle \dots \rangle_{\bar{V}} = \frac{\int \mathrm{d}\mathbf{q}\, e^{-\beta \bar{V}(\mathbf{q})} \dots}{\int \mathrm{d}\mathbf{q}\, e^{-\beta \bar{V}(\mathbf{q})}}$$

over the state-independent potential $\bar{V}(\mathbf{q})$ (cf. Eq. (S2)) as

$$\frac{P_+}{P_-} = \frac{Z_+}{Z_-} = \frac{\left\langle e^{-\beta(V_+(\mathbf{q}) - \bar{V}(\mathbf{q}))} \right\rangle_{\bar{V}}}{\left\langle e^{-\beta(V_-(\mathbf{q}) - \bar{V}(\mathbf{q}))} \right\rangle_{\bar{V}}} \tag{S27}$$

Using eqs. (S4) and (S5) this becomes (cf. Eq. (S25))

$$\frac{P_+}{P_-} = \frac{\left\langle e^{-\beta V_z(\mathbf{q})} \right\rangle_{\bar{V}}}{\left\langle e^{\beta V_z(\mathbf{q})} \right\rangle_{\bar{V}}} \tag{S28}$$

The probability of being in one particular state is

$$\begin{aligned}
\frac{P_+}{P_+ + P_-} &= \left(\frac{P_+ + P_-}{P_+} \right)^{-1} \\
&= \left(\frac{\left\langle e^{-\beta V_z(\mathbf{q})} \right\rangle_{\bar{V}} + \left\langle e^{+\beta V_z(\mathbf{q})} \right\rangle_{\bar{V}}}{\left\langle e^{-\beta V_z(\mathbf{q})} \right\rangle_{\bar{V}}} \right)^{-1} \\
&= \left(\frac{\langle 2\cosh(\beta V_z(\mathbf{q})) \rangle_{\bar{V}}}{\left\langle e^{-\beta V_z(\mathbf{q})} \right\rangle_{\bar{V}}} \right)^{-1} \\
\frac{P_+}{P_+ + P_-} &= \frac{\left\langle e^{-\beta V_z(\mathbf{q})} \right\rangle_{\bar{V}}}{2\, \langle \cosh(\beta V_z(\mathbf{q})) \rangle_{\bar{V}}}
\end{aligned} \tag{S29}$$

and thus

$$\frac{P_-}{P_+ + P_-} = \frac{\left\langle e^{\beta V_z(\mathbf{q})} \right\rangle_{\bar{V}}}{2\left\langle \cosh(\beta V_z(\mathbf{q})) \right\rangle_{\bar{V}}} \tag{S30}$$

Hence, the probability density as a function of only S_z is

$$\rho(S_z) = \frac{\left\langle e^{-\beta V_z(\mathbf{q})\,\mathrm{sign}(S_z)} \right\rangle_{\bar{V}}}{2\left\langle \cosh(\beta V_z(\mathbf{q})) \right\rangle_{\bar{V}}} \tag{S31}$$

Eq. (S31) is the identical result as obtained by Amati *et al.* in Eq. (26b) of[S1] (J. Chem. Phys. 159, 214114 (2023)).

For the free energy difference between the two states, one obtains

$$\Delta F = -k_{\mathrm{B}} T \ln \left(\frac{\left\langle e^{-\beta V_z(\mathbf{q})} \right\rangle_{\bar{V}}}{\left\langle e^{\beta V_z(\mathbf{q})} \right\rangle_{\bar{V}}} \right) = -k_{\mathrm{B}} T \ln \left(\frac{\int_0^1 \rho(S_z)\, \mathrm{d}S_z}{\int_{-1}^0 \rho(S_z)\, \mathrm{d}S_z} \right) \tag{S32}$$

S1.4 Fokker–Planck equation for $(\mathbf{q}, \mathbf{p}, \vec{S})$

Combining eqs. (S10), (S11), and (S16) we can write the Langevin dynamics as drift-diffusion process in the general form

$$\mathrm{d}\mathbf{\Gamma}_t = \boldsymbol{\mu}(\mathbf{\Gamma}_t, t)\,\mathrm{d}t + \boldsymbol{\sigma}(\mathbf{\Gamma}_t, t)\,\mathrm{d}\mathbf{W}_t \tag{S33}$$

Here $\mathbf{\Gamma}$ is the state vector

$$\mathbf{\Gamma} = \begin{pmatrix} \vdots \\ q_i \\ \vdots \\ p_i \\ \vdots \\ S_x \\ S_y \\ S_z \end{pmatrix} \tag{S34}$$

$\boldsymbol{\mu}$ denotes the drift vector

$$\boldsymbol{\mu}(\mathbf{\Gamma}) = \begin{pmatrix} \vdots \\ p_i/m_i \\ \vdots \\ -\frac{\partial_i \bar{V}}{\partial q_i} - \frac{\partial_i V_z}{\partial q_i}\,\mathrm{sign}(S_z) + 4V_z d_i S_x \delta(S_z) - \gamma p_i/m_i \\ \vdots \\ (\vec{H} \times \vec{S})_x \\ (\vec{H} \times \vec{S})_y \\ (\vec{H} \times \vec{S})_z \end{pmatrix} \tag{S35}$$

and $\boldsymbol{\sigma}$ the $(2N+3) \times N$ noise matrix; in block structure with the $\mathbf{q}$ block ($N \times N$ matrix) being 0, the $\mathbf{p}$ block ($N \times N$ matrix) being the noise, and the S block (3×3 matrix) being 0:

$$\boldsymbol{\sigma} = \begin{pmatrix} \mathbf{0} \\ \sqrt{2\gamma k_B T}\,\mathbb{I}_n \\ \mathbf{0} \end{pmatrix} \tag{S36}$$

and $\mathbf{W}_t$ the N-dimensional vector of the Wiener process, such that the Wiener increment is:

$$\boldsymbol{\sigma}\,\mathrm{d}\mathbf{W}_t = \begin{pmatrix} \vdots \\ 0 \\ \vdots \\ \sqrt{2\gamma k_B T}\,dW_i \\ \vdots \\ 0 \\ 0 \\ 0 \end{pmatrix} \tag{S37}$$

The corresponding diffusion tensor $\mathbf{D}$ is given by

$$\mathbf{D} = \frac{1}{2}\boldsymbol{\sigma}\boldsymbol{\sigma}^T \tag{S38}$$

where the components are

$$D_{ij}(\boldsymbol{\Gamma}) = \frac{1}{2}\sum_{k=1}^{N} \sigma_{ik}(\boldsymbol{\Gamma})\,\sigma_{jk}(\boldsymbol{\Gamma}) \tag{S39}$$

Due to the block structure of $\boldsymbol{\sigma}$ (cf. Eq. (S36)), the diffusion tensor $\mathbf{D}$ has a similar structure and can be written in the form of a $(2N+3)\times(2N+3)$ matrix as

$$\mathbf{D} = \frac{1}{2}\begin{pmatrix} \mathbf{0} \\ \sqrt{2\gamma k_B T}\,\mathbb{I}_n \\ \mathbf{0} \end{pmatrix}\Big(\mathbf{0}, \sqrt{2\gamma k_B T}\,\mathbb{I}_n, \mathbf{0}\Big) = \frac{1}{2}\begin{pmatrix} \mathbf{0} & \mathbf{0} & \mathbf{0} \\ \mathbf{0} & 2\gamma k_B T\,\mathbb{I}_n & \mathbf{0} \\ \mathbf{0} & \mathbf{0} & \mathbf{0} \end{pmatrix} = \begin{pmatrix} \mathbf{0} & \mathbf{0} & \mathbf{0} \\ \mathbf{0} & \gamma k_B T\,\mathbb{I}_n & \mathbf{0} \\ \mathbf{0} & \mathbf{0} & \mathbf{0} \end{pmatrix} \tag{S40}$$

Let $\rho(\boldsymbol{\Gamma}, t)$ be the probability density in $\boldsymbol{\Gamma}$-space and its time derivative

$$\frac{\partial \rho}{\partial t} = -\sum_{i=1}^{l} \frac{\partial}{\partial \Gamma_i}\left[\mu_i(\boldsymbol{\Gamma}, t)\rho(\boldsymbol{\Gamma}, t)\right] + \sum_{i=1}^{l}\sum_{j=1}^{l} \frac{\partial^2}{\partial \Gamma_i \partial \Gamma_j}\left[D_{ij}\rho(\boldsymbol{\Gamma}, t)\right] \tag{S41}$$

with $l = 2N+3$. $\mathbf{D}$ has only diagonal non-zero elements and only the diagonal components of $\mathbf{D}$ corresponding to the momenta $\mathbf{p}$ are non-zero ($D_{ij} = \gamma k_B T\, \delta_{ij}$ for momentum indices $p_{i=j}$). Thus

$$\sum_{i=1}^{l}\sum_{j=1}^{l} \frac{\partial^2}{\partial\Gamma_i \partial\Gamma_j} \left[D_{ij}\rho(\mathbf{\Gamma},t)\right] = \sum_{i=1}^{N} \frac{\partial^2}{\partial p_i^2} \left[\gamma k_B T\, \rho(\mathbf{\Gamma},t)\right] \tag{S42}$$

Expanding drift terms, we obtain:

$$\begin{aligned} -\sum_{i=1}^{l} \frac{\partial}{\partial \Gamma_i} \left[\mu_i(\mathbf{\Gamma},t)\rho(\mathbf{\Gamma},t)\right] =& -\sum_{i=1}^{N} \frac{\partial}{\partial q_i} \left[\frac{p_i}{m_i}\rho(\mathbf{\Gamma},t)\right] \\ & -\sum_{i=1}^{N} \frac{\partial}{\partial p_i} \left[\left(-\frac{\partial \bar{V}}{\partial q_i} - \frac{\partial V_z}{\partial q_i}\mathrm{sign}(S_z) + 4V_z d_i S_x \delta(S_z) - \frac{\gamma}{m_i}p_i\right)\rho(\mathbf{\Gamma},t)\right] \\ & -\sum_{\alpha=1}^{3} \frac{\partial}{\partial S_\alpha} \left[(\vec{H}\times\vec{S})_\alpha\, \rho(\mathbf{\Gamma},t)\right] \\ =& -\sum_{i=1}^{N} \frac{p_i}{m_i}\frac{\partial \rho}{\partial q_i} - \sum_{i=1}^{N} \left(-\frac{\partial \bar{V}}{\partial q_i} - \frac{\partial V_z}{\partial q_i}\mathrm{sign}(S_z) + 4V_z d_i S_x \delta(S_z) - \frac{\gamma}{m_i}p_i\right)\frac{\partial \rho}{\partial p_i} \\ & +\sum_{i=1}^{N} \frac{\gamma}{m_i}\frac{\partial}{\partial p_i}\left[p_i\rho\right] - \sum_{\alpha=1}^{3} \frac{\partial}{\partial S_\alpha} \left[(\vec{H}\times\vec{S})_\alpha\, \rho\right] \end{aligned} \tag{S43}$$

where for the indices in the spin terms, we for simplicity use the notation $\alpha = 1, 2, 3$ equivalent to $\alpha = x, y, z$. Inspecting the last cross-product term closer we find

$$\sum_\alpha \frac{\partial}{\partial S_\alpha}\left[(\vec{H}\times\vec{S})_\alpha \rho\right] = \sum_\alpha \left[\frac{\partial}{\partial S_\alpha}(\vec{H}\times\vec{S})_\alpha\right]\rho + (\vec{H}\times\vec{S})_\alpha \frac{\partial \rho}{\partial S_\alpha} \tag{S44}$$

Due to the property of the cross-product (cf. Eq. (S17)) we can see that S_α does not appear in the element $(\vec{H}\times\vec{S})_\alpha$, the first term of Eq. (S44) becomes zero, such that we can simplify

$$\sum_\alpha \frac{\partial}{\partial S_\alpha}\left[(\vec{H}\times\vec{S})_\alpha \rho\right] = \sum_\alpha (\vec{H}\times\vec{S})_\alpha \frac{\partial \rho}{\partial S_\alpha} \tag{S45}$$

Combining eqs. (S45), (S43) and (S42) with Eq. (S41), we obtain the Fokker-Planck equation

$$\frac{\partial \rho}{\partial t} = -\sum_{i=1}^{N} \frac{p_i}{m_i}\frac{\partial \rho}{\partial q_i} - \sum_{i=1}^{N}\left(-\frac{\partial \bar{V}}{\partial q_i} - \frac{\partial V_z}{\partial q_i}\text{sign}(S_z) + 4V_z d_i S_x \delta(S_z)\right)\frac{\partial \rho}{\partial p_i}$$
$$+ \gamma \sum_{i=1}^{N} \frac{\partial}{\partial p_i}\left(\frac{p_i}{m_i}\,\rho\right) - \sum_{\alpha=1}^{3}(\vec{H}\times\vec{S})_\alpha \frac{\partial \rho}{\partial S_\alpha} + \gamma k_B T \sum_{i=1}^{N}\frac{\partial^2 \rho}{\partial p_i^2} \tag{S46}$$

We now check whether the normalized probability density function

$$\rho(\mathbf{\Gamma}) = \rho(\mathbf{q},\mathbf{p},\vec{S}) = \frac{1}{Q}\exp\left[-\beta\left(\sum_i \frac{p_i^2}{2m_i} + \bar{V}(\mathbf{q}) + V_z(\mathbf{q})\text{sign}(S_z)\right)\right]\delta(|\vec{S}|-1) \tag{S47}$$

(cf. Eq. (S18)) is stationary. To accomplish this we inspect the single terms in Eq. (S46) separately, i.e., we evaluate the right hand side of Eq. (S46) to show that the normalized probability density function is stationary. The second derivative of ρ with respect to the momenta is

$$\frac{\partial^2 \rho}{\partial p_i^2} = \frac{\partial}{\partial p_i}\frac{\partial \rho}{\partial p_i}$$
$$= \frac{\partial}{\partial p_i}\left[\exp\left[-\beta\left(\bar{V}(\mathbf{q}) + V_z(\mathbf{q})\text{sign}(S_z)\right)\right]\delta(|\vec{S}|-1)\exp\left[-\beta\sum_{j=1}^{N}\frac{p_j^2}{2m_j}\right]\times\left(-\frac{\beta}{m_i}2p_i\right)\right]$$
$$= \frac{\partial}{\partial p_i}\left(-\beta\frac{p_i}{m_i}\rho\right) \tag{S48}$$

Hence

$$\gamma k_B T \sum_{i=1}^{N}\frac{\partial^2 \rho}{\partial p_i^2} = \gamma k_B T \sum_{i=1}^{N}\frac{\partial}{\partial p_i}\left[-\beta\frac{p_i}{m_i}\rho\right] = -\gamma\sum_{i=1}^{N}\frac{1}{m_i}\frac{\partial}{\partial p_i}\left[p_i \rho\right] \tag{S49}$$

such that the terms

$$\gamma\sum_{i=1}^{N}\frac{\partial}{\partial p_i}\left(\frac{p_i}{m_i}\rho\right) \qquad \text{and} \qquad \gamma k_B T\sum_{i=1}^{N}\frac{\partial^2 \rho}{\partial p_i^2}$$

of Eq. (S46) cancel.

Next we consider the first two terms.

S1.4.1 Case “away from the equator” ($S_z \neq 0$)

Here $4V_z d_i S_x \delta(S_z) = 0$ such that the derivatives of ρ are:

$$\frac{\partial \rho}{\partial q_i} = \rho \left[-\beta \frac{\partial \bar{V}}{\partial q_i} - \beta \frac{\partial V_z}{\partial q_i} \mathrm{sign}(S_z) \right] \tag{S50}$$

$$\frac{\partial \rho}{\partial p_i} = -\beta \frac{p_i}{m_i} \rho \tag{S51}$$

Then the two terms of Eq. (S46)

$$\begin{aligned}
-\sum_i \frac{p_i}{m_i} \frac{\partial \rho}{\partial q_i} - \sum_i \left(-\frac{\partial \bar{V}}{\partial q_i} - \frac{\partial V_z}{\partial q_i} \mathrm{sign}(S_z) \right) \frac{\partial \rho}{\partial p_i} =& -\sum_i \frac{p_i}{m_i} \rho \left(-\beta \frac{\partial \bar{V}}{\partial q_i} - \beta \frac{\partial V_z}{\partial q_i} \mathrm{sign}(S_z) \right) \\
&+ \sum_i \left(-\beta \frac{\partial \bar{V}}{\partial q_i} - \beta \frac{\partial V_z}{\partial q_i} \mathrm{sign}(S_z) \right) \left(-\frac{p_i}{m_i} \rho \right) \\
=& 0
\end{aligned}$$

cancel.

Hence, we are left with only a single term on the right hand side (RHS) of Eq. (S46).

$$\mathrm{RHS} = \sum_{\alpha=1}^{3} (\vec{H} \times \vec{S})_\alpha \frac{\partial \rho}{\partial S_\alpha}$$

Away from the equator the only $\vec{S}$-dependence of ρ is in the factor $\delta(|\vec{S}| - 1)$. By writing $\rho(\mathbf{q}, \mathbf{p}, \vec{S}) = g_0(\mathbf{q}, \mathbf{p}) \delta(|\vec{S}| - 1)$ we get:

$$\begin{aligned}
\sum_{\alpha=1}^{3} (\vec{H} \times \vec{S})_\alpha \frac{\partial \rho}{\partial S_\alpha} &= \sum_{\alpha=1}^{3} (\vec{H} \times \vec{S})_\alpha \; g_0(\mathbf{q}, \mathbf{p}) \frac{\partial \; \delta(|\vec{S}| - 1)}{\partial |\vec{S}|} \frac{\partial |\vec{S}|}{\partial S_\alpha} \\
&= g_0(\mathbf{q}, \mathbf{p}) \frac{\partial \; \delta(|\vec{S}| - 1)}{\partial |\vec{S}|} \sum_\alpha (\vec{H} \times \vec{S})_\alpha \frac{\partial |\vec{S}|}{\partial S_\alpha} \\
&= g_0(\mathbf{q}, \mathbf{p}) \frac{\partial \; \delta(|\vec{S}| - 1)}{\partial |\vec{S}|} \frac{1}{|\vec{S}|} \sum_\alpha (\vec{H} \times \vec{S})_\alpha \; S_\alpha \\
&= g_0(\mathbf{q}, \mathbf{p}) \frac{\partial \; \delta(|\vec{S}| - 1)}{\partial |\vec{S}|} \frac{1}{|\vec{S}|} \left[(\vec{H} \times \vec{S}) \cdot \vec{S} \right]
\end{aligned}$$

But $(\vec{H} \times \vec{S}) \perp \vec{S}$ such that

$$(\vec{H} \times \vec{S}) \cdot \vec{S} = 0 \tag{S52}$$

Thus

$$\sum_{\alpha=1}^{3} (\vec{H} \times \vec{S})_\alpha \frac{\partial \rho}{\partial S_\alpha} = 0 \tag{S53}$$

This holds for any distribution ρ depending only on $|\vec{S}|$. Therefore, away from the equator, the RHS = 0, and ρ is stationary.

S1.4.2 Case "at the equator" ($S_z = 0$)

At the equator, all previously considered terms cancel, but we get two extra contributions:

$$\text{RHS} = -\sum_{i=1}^{N} 4V_z(\mathbf{q}) d_i(\mathbf{q}) S_x \, \delta(S_z) \, \frac{\partial \rho}{\partial p_i} - \sum_{\alpha=1}^{3} (\vec{H} \times \vec{S})_\alpha \frac{\partial \rho}{\partial S_\alpha} \tag{S54}$$

From what we have derived in the previous subsection we can write the first term as

$$-\sum_{i=1}^{N} 4V_z(\mathbf{q}) d_i(\mathbf{q}) S_x \, \delta(S_z) \, \frac{\partial \rho}{\partial p_i} = \beta \sum_{i=1}^{N} 4V_z(\mathbf{q}) d_i(\mathbf{q}) S_x \, \delta(S_z) \, \frac{p_i}{m_i} \rho \tag{S55}$$

Inspecting the derivative of the probability density function with respect to the components of the spin vector, we find

$$\begin{aligned}
\frac{\partial \rho}{\partial S_\alpha} =& \frac{1}{Q} \exp\left[-\beta \left(\sum_i \frac{p_i^2}{2m_i} + \bar{V}(\mathbf{q}) + V_z(\mathbf{q})\text{sign}(S_z)\right)\right] \frac{\partial}{\partial S_\alpha} \delta(|\vec{S}| - 1) \\
&+ \frac{1}{Q} \exp\left[-\beta \left(\sum_i \frac{p_i^2}{2m_i} + \bar{V}(\mathbf{q}) + V_z(\mathbf{q})\text{sign}(S_z)\right)\right] \delta(|\vec{S}| - 1) \left(-\beta V_z(\mathbf{q}) \frac{\partial \, \text{sign}(S_z)}{\partial S_\alpha}\right) \\
=& \frac{1}{Q} \exp\left[-\beta \left(\sum_i \frac{p_i^2}{2m_i} + \bar{V}(\mathbf{q}) + V_z(\mathbf{q})\text{sign}(S_z)\right)\right] \frac{\partial \delta(|\vec{S}| - 1)}{\partial |\vec{S}|} \frac{S_\alpha}{|\vec{S}|} \\
&- \beta \rho V_z(\mathbf{q}) \frac{\partial \, \text{sign}(S_z)}{\partial S_z} \delta_{\alpha z}
\end{aligned}$$

The first term gives a contribution $(\vec{H} \times \vec{S}) \cdot \vec{S} = 0$ as before. Only the second term warrants

further inspection:

$$\sum_{\alpha=1}^{3}(\vec{H}\times\vec{S})_\alpha \frac{\partial\rho}{\partial S_\alpha} = -\beta\rho V_z(\mathbf{q})\ (\vec{H}\times\vec{S})_z\ \frac{\partial\,\mathrm{sign}(S_z)}{\partial S_z} \tag{S56}$$

Using

$$\frac{\partial}{\partial S_z}\mathrm{sign}(S_z) = 2\delta(S_z), \tag{S57}$$

and

$$(\vec{H}\times\vec{S})_z = -2S_x\sum_{i}^{N}\frac{p_i}{m_i}d_i(\mathbf{q}) \tag{S58}$$

we obtain

$$\begin{aligned}\sum_{\alpha=1}^{3}(\vec{H}\times\vec{S})_\alpha \frac{\partial\rho}{\partial S_\alpha} &= -2\beta\rho V_z(\mathbf{q})\delta(S_z)\left(-2S_x\sum_{i=1}^{N}\frac{p_i}{m_i}d_i(\mathbf{q})\right)\\ &= 4\beta\rho V_z(\mathbf{q})S_x\left(\sum_{i=1}^{N}\frac{p_i}{m_i}d_i(\mathbf{q})\right).\end{aligned}$$

Hence we get for the entire RHS:

$$\mathrm{RHS} = 4\beta\rho V_z(\mathbf{q})S_x\left(\sum_{i}^{N}\frac{p_i}{m_i}d_i(\mathbf{q})\right) - 4\beta\rho V_z(\mathbf{q})S_x\left(\sum_{i}^{N}\frac{p_i}{m_i}d_i(\mathbf{q})\right) \tag{S59}$$

$$= 0 \tag{S60}$$

Hence, we have shown that ρ (Eq. (S18) or (S47)) is the stationary solution of the Fokker-Planck equation (Eq. (S46)).

S2 Phase space volume conservation (for $\gamma = 0$)

The volume in $(\mathbf{q}, \mathbf{p}, \vec{S})$–space (or $\mathbf{\Gamma}$-space) is conserved if

$$\sum_{i=1}^{N} \frac{\partial \dot{q}_i}{\partial q_i} + \sum_{i=1}^{N} \frac{\partial \dot{p}_i}{\partial p_i} + \sum_{\alpha=1}^{3} \frac{\partial \dot{S}_\alpha}{\partial S_\alpha} = 0 \tag{S61}$$

Since

$$\dot{q}_i = \frac{p_i}{m_i}$$

(cf. Eq. (S10)) we have

$$\sum_i \frac{\partial \dot{q}_i}{\partial q_i} = \sum_i \frac{\partial}{\partial q_i}\left(\frac{p_i}{m}\right) = 0 \tag{S62}$$

Next, with

$$\dot{p}_i = -\frac{\partial \bar{V}}{\partial q_i} - \frac{\partial V_z}{\partial q_i}\,\mathrm{sign}(S_z) \tag{S63}$$

which contains no dependence on p_i, we get

$$\sum_i \frac{\partial \dot{p}_i}{\partial p_i} = 0 \tag{S64}$$

Thus, the dynamics in $(\mathbf{q}, \mathbf{p})$ conserve phase-space volume.

Now, we consider the spin part:

$$\dot{S}_\alpha = (\vec{H} \times \vec{S})_\alpha$$

such that

$$\sum_\alpha \frac{\partial \dot{S}_\alpha}{\partial S_\alpha} = \sum_\alpha \frac{\partial}{\partial S_\alpha}\left((\vec{H} \times \vec{S})_\alpha\right)$$

Using the fully antisymmetric tensor ϵ

$$(\vec{H} \times \vec{S})_\alpha = \sum_{\rho,\nu} \epsilon_{\alpha\rho\nu} H_\rho S_\nu, \tag{S65}$$

we can expand the derivative as

$$\sum_{\alpha} \frac{\partial}{\partial S_\alpha} (\vec{H} \times \vec{S})_\alpha = \sum_{\alpha,\rho,\nu} \epsilon_{\alpha\rho\nu} H_\rho \frac{\partial S_\nu}{\partial S_\alpha} \tag{S66}$$

Since

$$\frac{\partial S_\nu}{\partial S_\alpha} = \delta_{\alpha\nu}, \tag{S67}$$

we get

$$\sum_{\alpha,\rho,\nu} \epsilon_{\alpha\rho\nu} H_\rho \delta_{\alpha\nu} = \sum_{\alpha,\rho} \epsilon_{\alpha\rho\alpha} H_\rho = 0 \tag{S68}$$

because $\epsilon_{\alpha\rho\alpha} = 0$. Thus,

$$\sum_{\alpha} \frac{\partial \dot{S}_\alpha}{\partial S_\alpha} = 0. \tag{S69}$$

And we can conclude that

$$\sum_i \frac{\partial \dot{q}_i}{\partial q_i} + \sum_i \frac{\partial \dot{p}_i}{\partial p_i} + \sum_\alpha \frac{\partial \dot{S}_\alpha}{\partial S_\alpha} = 0, \tag{S70}$$

which means that the **phase space volume is conserved**.

S3 Inverting MASH Dynamics

S3.1 MASH equations of motion

Here we repeat the equations of motion of MASH dynamics for an easier overview:

$$\dot{q}_i = \frac{p_i}{m_i}$$

$$\dot{p}_i = -\frac{\partial \bar{V}}{\partial q_i} - \frac{\partial V_z}{\partial q_i}\,\text{sign}(S_z) + 4V_z(\mathbf{q})d_i(\mathbf{q})S_x\delta(S_z) - \frac{\gamma}{m_i}p_i + \mathcal{F}_i(t)$$

$$\dot{\vec{S}} = \vec{H}(\mathbf{q},\mathbf{p}) \times \vec{S}, \qquad \vec{H} = \begin{pmatrix} 0 \\ 2\sum_{i=1}^{N} d_i(\mathbf{q})\frac{p_i}{m_i} \\ 2V_z(\mathbf{q}) \end{pmatrix}$$

$$\dot{\vec{S}} = \begin{pmatrix} 2\sum_{i=1}^{N} d_i(\mathbf{q})\frac{p_i}{m_i}S_z - 2V_z(\mathbf{q})S_y \\ 2V_z(\mathbf{q})S_x \\ -2\sum_{i=1}^{N} d_i(\mathbf{q})\frac{p_i}{m_i}S_x \end{pmatrix}$$

S3.2 Interpretation of time reversal

In configuration space, we want trajectories to retrace when momenta are inverted. Thus, any variable that appears “like a coordinate” should not change sign under time reversal.

For that to happen, S_z should retrace its trajectory as well. Since it appears in the potential energy and in the force, it should not change sign upon time inversion. It behaves like a ”position-like” variable.

The equation of motion for S_z is

$$\dot{S}_z = -q\sum_i d_i(\mathbf{q})\frac{p_i}{m_i}\,S_x \tag{S71}$$

So if the momenta are inverted, $\dot{S}_z$ changes sign (it needs to do that for the trajectory to go back in time) only if S_x does not change its sign. Therefore, S_x is a position-like (sign does not flip) variable as well.

For the right hand side of the equation of motion of S_x to invert sign:

$$\dot{S}_x = \frac{2}{m}\sum_i d_i(\mathbf{q})p_i\,S_z - V_z(\mathbf{q})S_y \tag{S72}$$

S_y needs to be a momentum-like variable, *i.e.*, S_y changes sign if we move back in time.

This is consistent with the equation for S_y:

$$\dot{S}_y = 2V_z(\mathbf{q})S_x \tag{S73}$$

The RHS does **not** change sign under time reversal, as required for the equation of motion of a momentum-like variable.

S3.3 Summary of Time Inversion Transformations

$$\begin{aligned} q_i &\to q_i \\ p_i &\to -p_i \end{aligned}$$

$$\begin{aligned} S_x &\to S_x \\ S_y &\to -S_y \\ S_z &\to S_z \end{aligned}$$

S4 Detailed Balance

Let $\mathbf{\Gamma}$ be the state vector and $\bar{\mathbf{\Gamma}}$ its inverted counterpart.

$$\mathbf{\Gamma} = \begin{pmatrix} \vdots \\ q_i \\ \vdots \\ p_i \\ \vdots \\ S_x \\ S_y \\ S_z \end{pmatrix}, \qquad \bar{\mathbf{\Gamma}} = \begin{pmatrix} \vdots \\ q_i \\ \vdots \\ -p_i \\ \vdots \\ S_x \\ -S_y \\ S_z \end{pmatrix}. \tag{S74}$$

Here, q_i, S_x, S_z are even under time reversal, and p_i, S_y are odd.

The condition for detailed balance is

$$\rho(\mathbf{\Gamma_1})\, P(\mathbf{\Gamma}_1 \rightarrow \mathbf{\Gamma}_2) = \rho(\bar{\mathbf{\Gamma}}_2)\, P(\bar{\mathbf{\Gamma}}_2 \rightarrow \bar{\mathbf{\Gamma}}_1) \tag{S75}$$

with probability distribution ρ and transition rates P. As a reminder, the stationary distribution is

$$\rho(\mathbf{\Gamma}) = Q^{-1} \exp\left[-\beta\left(\sum_i \frac{p_i^2}{2m_i} + \bar{V}(\mathbf{q}) + V_z(\mathbf{q})\,\mathrm{sign}(S_z)\right)\right] \delta(|\vec{S}| - 1)$$

and the Fokker-Planck equation

$$\frac{\partial f}{\partial t} = -\sum_{i=1}^{m} \frac{\partial}{\partial \Gamma_i}\left[\mu_i(\mathbf{\Gamma}, t) f(\mathbf{\Gamma}, t)\right] + \sum_{i=1}^{m}\sum_{j=1}^{m} \frac{\partial^2}{\partial X_i \partial X_j}\left[D_{ij} f(\mathbf{\Gamma}, t)\right]\ ,$$

with drift vector $\boldsymbol{\mu}$ and diffusion matrix $\mathbf{D}$ defined in Eq. (S35) and (S40), respectively.

S4.1 Time-reversal parity operator

Introduce ε_i such that

$$\varepsilon_i = \begin{cases} +1, & \Gamma_i \text{ even under time reversal,} \\ -1, & \Gamma_i \text{ odd under time reversal.} \end{cases} \tag{S76}$$

and

$$\bar{\mathbf{\Gamma}} = \varepsilon \mathbf{\Gamma} = \begin{pmatrix} \varepsilon_1 \Gamma_1 \\ \varepsilon_2 \Gamma_2 \\ \vdots \\ \varepsilon_m \Gamma_m \end{pmatrix} . \tag{S77}$$

Using the parity operator, the detailed balance expression becomes

$$\rho(\mathbf{\Gamma}_1)\, p(\mathbf{\Gamma}_1 \to \mathbf{\Gamma}_2) = \rho(\varepsilon\mathbf{\Gamma}_2)\, p(\varepsilon\mathbf{\Gamma}_2 \to \varepsilon\mathbf{\Gamma}_1). \tag{S78}$$

For our probability density function ρ we have

$$\rho(\mathbf{\Gamma}) = \rho(\varepsilon\mathbf{\Gamma}) \ , \tag{S79}$$

which means that the stationary distribution is invariant under time reversal.

S4.2 Conditions for Detailed Balance

The following conditions are necessary and sufficient for detailed balance:[S2]

$$\varepsilon_i \mu_i(\varepsilon\mathbf{\Gamma})\, \rho(\mathbf{\Gamma}) = -\mu_i(\mathbf{\Gamma})\rho(\mathbf{\Gamma}) + \sum_j \frac{\partial}{\partial \Gamma_j} \left(2 D_{ij}\, \rho(\mathbf{\Gamma})\right) \tag{S80}$$

$$\varepsilon_i \varepsilon_j\, D_{ij}(\varepsilon\mathbf{\Gamma}) = D_{ij}(\mathbf{\Gamma}) \tag{S81}$$

S4.3 Check condition Eq. (S80)

As a reminder the elements of the time-reversal parity operator are:

$$\varepsilon_i = \begin{cases} +1, & i = 1, \ldots, N \quad \text{(positions: even)} \\ -1, & i = N+1, \ldots, 2N \quad \text{(momenta: odd)} \\ +1, & i = 2N+1, 2N+3 \quad \text{(spin } S_x, S_z \text{ even)} \\ -1, & i = 2N+2 \quad \text{(spin } S_y \text{ odd)} \end{cases}$$

S4.3.1 Case 1: positions ($i = 1, \ldots, N$, $\varepsilon_i = 1$)

For the elements of $\boldsymbol{\mu}$ corresponding to the position indices, we have

$$\mu_i = \frac{p_i}{m_i} \quad \text{such that} \quad \varepsilon_i \mu_i(\varepsilon \boldsymbol{\Gamma}) = -\frac{p_i}{m_i} \ .$$

For these indices, diffusion does not contribute anything:

$$\sum_j \frac{\partial}{\partial \Gamma_j} (D_{ij} \rho(\boldsymbol{\Gamma})) = 0 \tag{S82}$$

Thus

$$\varepsilon_i \mu_i(\varepsilon \boldsymbol{\Gamma}) \rho(\boldsymbol{\Gamma}) = -\frac{p_i}{m_i} \rho(\boldsymbol{\Gamma}) = -\mu_i(\boldsymbol{\Gamma}) \rho(\boldsymbol{\Gamma}), \tag{S83}$$

and the condition is satisfied for coordinates.

S4.3.2 Case 2: momenta ($i = N+1, \ldots, 2N$, $\varepsilon_i = -1$)

For these indices, the drift vector has the values

$$\mu_i = -\frac{\partial \bar{V}}{\partial q_{i-N}} - \frac{\partial V_z}{\partial q_{i-N}} \text{sign}(S_z) + 4 V_z d_{i-N} S_x \delta(S_z) - \frac{\gamma}{m_{i-N}} p_{i-N} \tag{S84}$$

Under time reversal of the vector, we obtain

$$\varepsilon_i\mu_i(\varepsilon\mathbf{\Gamma})\rho(\mathbf{\Gamma}) = -\left(-\frac{\partial\bar{V}}{\partial q_{i-N}} - \frac{\partial V_z}{\partial q_{i-N}}\mathrm{sign}(S_z) + 4V_z d_{i-N} S_x \delta(S_z) + \frac{\gamma}{m_{i-N}} p_{i-N}\right)\rho(\mathbf{\Gamma})$$

and

$$-\mu_i(\mathbf{\Gamma})\rho(\mathbf{\Gamma}) = -\left(-\frac{\partial\bar{V}}{\partial q_{i-N}} - \frac{\partial V_z}{\partial q_{i-N}}\mathrm{sign}(S_z) + 4V_z d_{i-N} S_x \delta(S_z) - \frac{\gamma}{m_{i-N}} p_{i-N}\right)\rho(\mathbf{\Gamma})$$

such that

$$\varepsilon_i\mu_i(\varepsilon\mathbf{\Gamma})\rho(\mathbf{\Gamma}) + \mu_i(\mathbf{\Gamma})\rho(\mathbf{\Gamma}) = -2\gamma\frac{p_{i-N}}{m_{i-N}}\,\rho(\mathbf{\Gamma}) \tag{S85}$$

As a reminder, the elements of the diffusion matrix are:

$$D_{ij} = \begin{cases} \gamma k_B T\,\delta_{ij}, & j = N+1,\dots,2N \\ 0, & \text{else} \end{cases}$$

The derivative of the diffusion term in Eq. (S80) for the momenta indices is therefore

$$\sum_j \frac{\partial}{\partial\Gamma_j}\left(2D_{ij}\rho(\mathbf{\Gamma})\right) = 2\gamma k_B T \sum_{j=N+1}^{2N} \frac{\partial\rho(\mathbf{\Gamma})}{\partial p_{j-N}}\delta_{ij} = -2\beta\gamma k_B T\frac{p_{i-N}}{m_{i-N}}\rho(\mathbf{\Gamma}) = -2\gamma\frac{p_{i-N}}{m_{i-N}}\,\rho(\mathbf{\Gamma}). \tag{S86}$$

Hence, condition Eq. (S80) is also fulfilled for the momenta.

S4.3.3 Case 3: Spin components ($i = 2N+1,\ 2N+2,\ 2N+3$)

For spin degrees of freedom we have $D_{ij} = 0$ (cf. Eq. (S40). Thus we only must check:

$$\varepsilon_i\mu_i(\varepsilon\mathbf{\Gamma})\rho(\mathbf{\Gamma}) = -\mu_i(\mathbf{\Gamma})\rho(\mathbf{\Gamma})$$

For the left hand side, we have

$$\varepsilon_i \mu_i(\varepsilon\mathbf{\Gamma})\rho(\mathbf{\Gamma}) = \begin{pmatrix} 1\dot{S}_x(\varepsilon\mathbf{\Gamma}) \\ -1\dot{S}_y(\varepsilon\mathbf{\Gamma}) \\ 1\dot{S}_z(\varepsilon\mathbf{\Gamma}) \end{pmatrix} \rho(\mathbf{\Gamma}) = \begin{pmatrix} -2\sum_i d_i(\mathbf{q})\frac{p_i}{m_i}S_z + 2V_z(\mathbf{q})S_y \\ -2V_z(\mathbf{q})S_x \\ +2\sum_i d_i(\mathbf{q})\frac{p_i}{m_i}S_x \end{pmatrix} \rho(\mathbf{\Gamma}) \tag{S87}$$

and for the right hand side

$$-\mu_i(\mathbf{\Gamma})\rho(\mathbf{\Gamma}) = -\begin{pmatrix} 1\dot{S}_x(\mathbf{\Gamma}) \\ 1\dot{S}_y(\mathbf{\Gamma}) \\ 1\dot{S}_z(\mathbf{\Gamma}) \end{pmatrix} \rho(\mathbf{\Gamma}) = -\begin{pmatrix} 2\sum_i d_i(\mathbf{q})\frac{p_i}{m_i}S_z - 2V_z(\mathbf{q})S_y \\ +2V_z(\mathbf{q})S_x \\ -2\sum_i d_i(\mathbf{q})\frac{p_i}{m_i}S_x \end{pmatrix} \rho(\mathbf{\Gamma}) \tag{S88}$$

Thus, the condition holds for all spin degrees of freedom as well.

S4.4 Check condition Eq. (S81)

For the diffusion matrix, we have:

$$\varepsilon_i \varepsilon_j \, D_{ij}(\varepsilon\mathbf{\Gamma}) = D_{ij}(\mathbf{\Gamma}) \tag{S89}$$

This is true, because only the diagonal elements for the momentum indices are non-zero and constant.

$$D = \begin{pmatrix} 0 & 0 & 0 \\ 0 & \gamma k_B T \mathbb{I}_n & 0 \\ 0 & 0 & 0 \end{pmatrix}$$

In other words, the nonzero entries only for momenta mean that

- indices $i, j \in \{N+1, \ldots, 2N\}$,
- for these: $\varepsilon_i = \varepsilon_j = -1$,
- thus $\varepsilon_i \varepsilon_j = +1$.

Such that for the momentum indices we have

$$\varepsilon_i \varepsilon_j D_{ij}(\varepsilon \mathbf{\Gamma}) = D_{ij}(\mathbf{\Gamma}). \tag{S90}$$

and everywhere else $D_{ij} = 0$, so the condition holds trivially. Thus, condition Eq. (S81) holds as well.

Hence, MASH dynamics satisfies detailed balance.

S5 Relation of the dynamics of $\vec{S}$ to that of c_+ and c_-

The wave function of a two-state system, can be written as

$$|\Psi\rangle = c_-|\psi_-(\mathbf{q})\rangle + c_+|\psi_+(\mathbf{q})\rangle \ , \tag{S91}$$

where the first term denotes the ground state and the second one denotes the excited state. In the adiabatic representation, the Schrödinger equation using the energy impression from Eq. (S1) leads to the following equations of motion for the coefficients c_- and c_+

$$\dot{c}_- = -\frac{i}{\hbar}c_-V_-(\mathbf{q}) - \dot{\mathbf{q}} \cdot \mathbf{F}(\mathbf{q})c_+ \tag{S92}$$

$$\dot{c}_+ = -\frac{i}{\hbar}c_+V_+(\mathbf{q}) + \dot{\mathbf{q}} \cdot \mathbf{F}(\mathbf{q})c_- \tag{S93}$$

where $\mathbf{F} = \langle\psi_-|\frac{\partial}{\partial\mathbf{q}}|\psi_+\rangle$ is the nonadiabatic coupling element. Note that $\mathbf{F} = -\mathbf{F}^*$.

In order to connect the equations of motion for the coefficients with those of the spin vector, the elements of the spin vector are related to the coefficients by:

$$S_x = 2\mathrm{Re}\left[c_+^*c_-\right] \tag{S94}$$

$$S_y = 2\mathrm{Im}\left[c_+^*c_-\right] \tag{S95}$$

$$S_z = |c_+|^2 - |c_-|^2 \tag{S96}$$

Taking the time derivatives

$$\begin{aligned}\dot{S}_x &= 2\frac{\mathrm{d}}{\mathrm{d}t}\mathrm{Re}\left[c_+^*c_-\right] = 2\frac{\mathrm{d}}{\mathrm{d}t}\frac{\left(c_+^*c_- + (c_+^*c_-)^*\right)}{2} = \frac{\mathrm{d}}{\mathrm{d}t}\left(c_+^*c_- + c_+c_-^*\right)\\ &= \dot{c}_+^*c_- + c_+^*\dot{c}_- + \dot{c}_+c-^* + c_+\dot{c}_-^*\\ &= 2\frac{\dot{c}_+^*c_- + c_+^*\dot{c}_- + \left(\dot{c}_+^*c_- + c_+^*\dot{c}_-\right)^*}{2}\\ &= 2\mathrm{Re}\left[\dot{c}_+^*c_- + c_+^*\dot{c}_-\right]\end{aligned} \tag{S97}$$

Similarly,

$$\dot{S}_y = 2\frac{\mathrm{d}}{\mathrm{d}t}\mathrm{Im}\left[c_+^* c_-\right] = 2\mathrm{Im}\left[\dot{c}_+^* c_- + c_+^* \dot{c}_-\right] \tag{S98}$$

and

$$\begin{aligned} \dot{S}_z &= \frac{\mathrm{d}}{\mathrm{d}t}\left(|c_+|^2 - |c_-|^2\right) = \frac{\mathrm{d}}{\mathrm{d}t}\left(c_+^* c_+ - c_-^* c_-\right) \\ &= \dot{c}_+^* c_+ + c_+^* \dot{c}_+ - \dot{c}_-^* c_- - c_-^* \dot{c}_- \ . \end{aligned} \tag{S99}$$

Next, we will evaluate the mixed products of coefficients and their time derivatives, and use the assumption that $\mathbf{F}$ is real:

$$\begin{aligned} \dot{c}_+^* c_- + c_+^* \dot{c}_- &= \left(-\frac{i}{\hbar} c_+ V_+ + \dot{\mathbf{q}}\cdot\mathbf{F}\ c_-\right)^* c_- + c_+^* \left(-\frac{i}{\hbar} c_- V_- - \dot{\mathbf{q}}\cdot\mathbf{F}\ c_+\right) \\ &= \frac{i}{\hbar} V_+ c_+^* c_- + \dot{\mathbf{q}}\cdot\mathbf{F}\ c_-^* c_- - \frac{i}{\hbar} V_- c_+^* c_- - \dot{\mathbf{q}}\cdot\mathbf{F}\ c_+^* c_+ \end{aligned}$$

Using Eq. (S3) we obtain

$$\dot{c}_+^* c_- + c_+^* \dot{c}_- = \frac{2i}{\hbar} V_z(q) c_+^* c_- - \dot{\mathbf{q}}\cdot\mathbf{F}\left(|c_+|^2 - |c_-|^2\right) \tag{S100}$$

The other products are

$$c_+^* \dot{c}_+ = c_+^* \left(-\frac{i}{\hbar} c_+ V_+ + \dot{\mathbf{q}}\cdot\mathbf{F}\ c_-\right) = -\frac{i}{\hbar} V_+ c_+^* c_+ + \dot{\mathbf{q}}\cdot\mathbf{F}\ c_+^* c_- = -\frac{i}{\hbar} V_+ |c_+|^2 + \dot{\mathbf{q}}\cdot\mathbf{F}\ c_+^* c_-$$

and

$$c_-^* \dot{c}_- = c_-^* \left(-\frac{i}{\hbar} c_- V_- - \dot{\mathbf{q}}\cdot\mathbf{F}\ c_+\right) = -\frac{i}{\hbar} V_- c_-^* c_- - \dot{\mathbf{q}}\cdot\mathbf{F}\ c_-^* c_+ = -\frac{i}{\hbar} V_- |c_-|^2 - \dot{\mathbf{q}}\cdot\mathbf{F}\ c_-^* c_+$$

Plugging the results into the expressions for $\vec{S}$ (cf. eqs. (S94), (S95) and (S96)), one obtains:

$$\begin{aligned}\dot{S}_x &= 2\mathrm{Re}\left[\frac{2i}{\hbar}V_z(q)c_+^*c_- - \dot{\mathbf{q}}\cdot\mathbf{F}\ \left(|c_+|^2-|c_-|^2\right)\right] \\ &= 4\frac{V_z}{\hbar}\mathrm{Re}\left(ic_+^*c_-\right) - 2\dot{\mathbf{q}}\cdot\mathbf{F}\ S_z = -2\frac{V_z}{\hbar}2\mathrm{Im}\left(c_+^*c_-\right) - 2\dot{\mathbf{q}}\cdot\mathbf{F}\ S_z \\ &= -2\frac{V_z}{\hbar}S_y - 2\dot{\mathbf{q}}\cdot\mathbf{F}\ S_z\end{aligned}$$

and

$$\begin{aligned}\dot{S}_y &= 2\mathrm{Im}\left[\frac{2i}{\hbar}V_z(q)c_+^*c_- - \dot{\mathbf{q}}\cdot\mathbf{F}\ \left(|c_+|^2-|c_-|^2\right)\right] \\ &= 2\mathrm{Im}\left[\frac{2i}{\hbar}V_z(q)c_+^*c_-\right] = 2\frac{V_z}{\hbar}\mathrm{Re}\left(c_+^*c_-\right) = 2\frac{V_z}{\hbar}S_x\end{aligned}$$

and

$$\dot{S}_z = 2\dot{\mathbf{q}}\cdot\mathbf{F}\ \mathrm{Re}(c_+^*c_-) + 2\dot{\mathbf{q}}\cdot\mathbf{F}\ \mathrm{Re}(c_+^*c_-) = 2\dot{\mathbf{q}}\cdot\mathbf{F}\ 2\mathrm{Re}(c_+^*c_-) = 2\dot{\mathbf{q}}\cdot\mathbf{F}\ S_x$$

Thus, we arrive at the equations of motion

$$\dot{S}_x = -\frac{2V_z(q)}{\hbar}S_y - 2\dot{\mathbf{q}}\cdot\mathbf{F}\ S_z \tag{S101}$$

$$\dot{S}_y = \frac{2V_z(q)}{\hbar}S_x \tag{S102}$$

$$\dot{S}_z = 2\dot{\mathbf{q}}\cdot\mathbf{F}\ S_x \tag{S103}$$

Eqs. (S101), (S102) and (S103) are identical to the equations of motion from Mannouch *et al.*[S3] This means that, alternatively to propagating the spin vector, one can use the coefficients c_+ and c_- instead.

S6 Reflection of $\vec{S}$ at frustrated transition

If there is a transition from the southern to the northern hemisphere of the Bloch sphere (or vice versa), or in the language of surface hopping a "hop", and the kinetic energy K is insufficient, $K < 2V_z$, the hop is frustrated. In this case, the Bloch vector $\vec{S}$ is reflected at the equator. We assume a reflection takes place between time steps t and $t+1$. To the first (linear) approximation, the vector $\vec{S}(t + \Delta t)$ is obtained by reflecting $\vec{S}'(t + \Delta t)$, i.e., the vector calculated without reflection on the equator:

$$
\begin{aligned}
S_x(t + \Delta t) &= S'_x(t + \Delta t) \\
S_y(t + \Delta t) &= S'_y(t + \Delta t) \\
S_z(t + \Delta t) &= -S'_z(t + \Delta t)
\end{aligned}
$$

Since we do not run the dynamics in $\vec{S}$, but rather in the coefficients c_+ and c_-, we need to implement the reflection in the coefficients c_+ and c_-. We write c'_+ and c'_- — the coefficients before the reflection — as

$$
\begin{aligned}
c'_+ &= r_+ e^{i\varphi_+} \\
c'_- &= r_- e^{i\varphi_-}
\end{aligned}
$$

The reflection is performed by exchanging the norms

$$
\begin{aligned}
c_+ &= r_- e^{i\varphi_+} = c'_+ \frac{r_-}{r_+} \\
c_- &= r_+ e^{i\varphi_-} = c'_- \frac{r_+}{r_-} \ .
\end{aligned}
$$

This changes $\vec{S}$ in the following way:

$$S_x = 2\mathrm{Re}[c_+^* c_-] = 2\mathrm{Re}\left[c_+'^* \frac{r_-}{r_+} c_-' \frac{r_+}{r_-}\right] = 2\mathrm{Re}[c_+'^* c_-'] = S_x' \tag{S104}$$

$$S_y = 2\mathrm{Im}[c_+^* c_-] = 2\mathrm{Im}\left[c_+'^* \frac{r_-}{r_+} c_-' \frac{r_+}{r_-}\right] = 2\mathrm{Im}[c_+'^* c_-'] = S_y' \tag{S105}$$

$$S_z = |c_+|^2 - |c_-|^2 = r_-^2 - r_+^2 = -(r_+^2 - r_-^2) = -S_z' \tag{S106}$$

Hence, exchanging the norms r_+ and r_- leaves S_x and S_y invariant, but changes the sign of S_z, exactly as intended.

In the reversible implementation of the MASH dynamics used in this work, however, whether a hop is frustrated can be known exactly when the spin vector crosses the equator. Therefore, the need to reflect S_z as a electronic variable readjustment vanishes. Once the nonadiabatic coupling vector component of velocity is reflected, $\dot{S}_z$ is inverted automatically, and this reflects the spin vector off the equator.

S7 Inversion of Coefficients

The following derivation is true for two or multiple pairwise-coupled electronic states. The total electronic wave function is written as a linear combination of electronic basis states $|\psi_i\rangle$:

$$|\Psi(t)\rangle = \sum_i c_i|\psi_i\rangle\,. \tag{S107}$$

While the electronic wave function spans all states, the classical nuclei evolve at each time step on a single *active* potential energy surface. In the unSMASH[S4] formulation of MASH, a generalization that treats more than two electronic states, the coupling between the active state and each of the remaining states are represented by $N_s - 1$ Bloch spheres, where N_s is the total number of electronic states.

For each Bloch sphere, the corresponding spin vector $\vec{S}$ can be obtained directly from the electronic amplitudes:

$$S_x = 2\,\mathrm{Re}[c^*_{\mathrm{act}}c_\lambda]\,, \tag{S108}$$

$$S_y = 2\,\mathrm{Im}[c^*_{\mathrm{act}}c_\lambda]\,, \tag{S109}$$

$$S_z = |c_{\mathrm{act}}|^2 - |c_\lambda|^2\,, \tag{S110}$$

where c_{act} denotes the coefficient of the active state and c_λ that of the coupled state. As shown in Section S3, under time inversion the components S_x and S_z remain unchanged, whereas S_y changes sign.

Because most nonadiabatic dynamics engines propagate only the coefficients c_i, time inversion of the Bloch sphere must be implemented through a transformation of the coefficients instead. Complex conjugation of all coefficients produces exactly the required transformation:

$$c_i \;\longrightarrow\; c_i^*\,, \tag{S111}$$

for all i.

S7.1 Proof

Each electronic coefficient can be written as

$$c_i = \mathrm{Re}[c_i] + i\,\mathrm{Im}[c_i]\,,$$

so that its complex conjugate is

$$c_i^* = \mathrm{Re}[c_i] - i\,\mathrm{Im}[c_i]\,.$$

The mixed product entering the Bloch-sphere components is

$$c_{\mathrm{act}}^* c_\lambda = \mathrm{Re}[c_{\mathrm{act}}]\,\mathrm{Re}[c_\lambda] + \mathrm{Im}[c_{\mathrm{act}}]\,\mathrm{Im}[c_\lambda] - i\,\mathrm{Im}[c_{\mathrm{act}}]\,\mathrm{Re}[c_\lambda] + i\,\mathrm{Re}[c_{\mathrm{act}}]\,\mathrm{Im}[c_\lambda]\,.$$

We now examine the three components individually.

$\boldsymbol{S_x}$: Since

$$S_x(c_{\mathrm{act}}, c_\lambda) \propto \mathrm{Re}[c_{\mathrm{act}}^* c_\lambda] = \mathrm{Re}[c_{\mathrm{act}}]\,\mathrm{Re}[c_\lambda] + \mathrm{Im}[c_{\mathrm{act}}]\,\mathrm{Im}[c_\lambda],$$

complex conjugation of all coefficients leaves S_x invariant:

$$\mathrm{Re}[(c_{\mathrm{act}}^*)^*(c_\lambda^*)] = \mathrm{Re}[c_{\mathrm{act}} c_\lambda^*] = \mathrm{Re}[c_{\mathrm{act}}]\,\mathrm{Re}[c_\lambda] + \mathrm{Im}[c_{\mathrm{act}}]\,\mathrm{Im}[c_\lambda] = \mathrm{Re}[c_{\mathrm{act}}^* c_\lambda] \propto S_x(c_{\mathrm{act}}, c_\lambda)\,.$$

$\boldsymbol{S_z}$: Because

$$S_z(c_{\mathrm{act}}, c_\lambda) = |c_{\mathrm{act}}|^2 - |c_\lambda|^2,$$

and the modulus is invariant under complex conjugation,

$$S_z(c_{\mathrm{act}}^*, c_\lambda^*) = |c_{\mathrm{act}}|^2 - |c_\lambda|^2 = S_z(c_{\mathrm{act}}, c_\lambda)\,.$$

$\boldsymbol{S_y}$: For the imaginary component we have

$$S_y(c_{\mathrm{act}}, c_\lambda) = 2\,\mathrm{Im}[c^*_{\mathrm{act}} c_\lambda]\,.$$

Under conjugation,

$$\mathrm{Im}[(c^*_{\mathrm{act}})^* c^*_\lambda] = \mathrm{Im}[c_{\mathrm{act}} c^*_\lambda] = -\,\mathrm{Im}[c^*_{\mathrm{act}} c_\lambda]\,,$$

which yields

$$S_y(c^*_{\mathrm{act}}, c^*_\lambda) = -\,S_y(c_{\mathrm{act}}, c_\lambda)\,.$$

Together, these relations demonstrate that complex conjugation of all coefficients produces the correct time-inversion transformation of the spin vector $\vec{S}$.

S8 Local Diabatization

The nuclear propagation in the MASH dynamics is performed using a half-step velocity-Verlet algorithm:

$$p(t+\frac{\Delta t}{2}) = p(t) + \frac{\Delta}{2}F(q(t)) \tag{S112}$$

$$q(t+\Delta t) = q(t) + \Delta t\frac{p(t+\Delta t)}{m} \tag{S113}$$

$$p(t+\Delta t) = p(t+\frac{\Delta t}{2}) + \frac{\Delta t}{2}F(q(t+\Delta t)), \tag{S114}$$

where the adiabatic force, $F(q(t))$, is defined as:

$$F_j(q(t)) = -\frac{\partial V_-(q)}{\partial q_j}h(-S_z) - \frac{\partial V_+(q)}{\partial q_j}h(S_z). \tag{S115}$$

The electronic coefficients are evolved using the local diabatization (LD) scheme,[S5] after which they are mapped onto the components of the spin vector $\vec{S}$. The propagation of the electronic coefficient vector is given by

$$\mathbf{c}(t+\Delta t) = \mathbf{T}^{-1}e^{-\frac{i}{2\hbar}(\mathbf{E}(t)+\tilde{\mathbf{H}}(t+\Delta t))\Delta t}\mathbf{c}(t)\ , \tag{S116}$$

where $\mathbf{T}$ is the Löwdin-orthogonalized overlap matrix between the adiabatic basis at times t and $t+\Delta t$:

$$\mathbf{T} = \mathbf{S}(\mathbf{S}^\dagger\mathbf{S})^{-\frac{1}{2}} \qquad \text{with} \qquad [\mathbf{S}]_{ij} = \langle\psi_i(t)|\psi_j(t+\Delta t)|\ \rangle, \tag{S117}$$

with $\psi_i(t)$ denoting the eigenvectors of the Hamiltonian $\mathbf{H}(q(t))$. The locally diabatic Hamiltonian is constructed from the diagonal adiabatic energy matrix $\mathbf{E}(t+\Delta t)$ according to

$$\tilde{\mathbf{H}}(t+\Delta t) = \mathbf{T}\mathbf{E}(t+\Delta t)\mathbf{T}^{-1}. \tag{S118}$$

S9 Numerical Accuracy with Naive Velocity-Verlet

To verify the practical time reversibility of the implementation, we performed a series of forward–backward propagation tests using three different initial conditions ($q = 1.0$ a.u., $\mathbf{c} = (1.0, 0.0)^\top$): (i) high kinetic energy ($v = -0.5$ a.u.), leading to multiple hops; (ii) moderate kinetic energy ($v = -0.05$ a.u.), producing fewer hops; and (iii) low kinetic energy ($v = -0.002$ a.u.), resulting in purely adiabatic dynamics. Trajectories without hops are perfectly reversible for both implementations. However, for trajectories involving electronic transitions, the naive velocity-Verlet implementation accumulates significant deviations after forward–backward propagation, as summarized in Table S1. In contrast, simulations employing the line-search procedure remain reversible up to machine precision.

The absolute percentage error (APE) quantifies the relative deviation between the initial phase-space point and the configuration obtained after propagating N steps forward and subsequently N steps backward. For reference, the initial coordinate is $q = 1.0$ a.u., so an error of 400% in the coordinate corresponds to an absolute deviation of roughly 4 a.u., indicating that the trajectory fails to return even approximately to the starting configuration. Similarly, for the moderate-velocity case ($v = -0.05$ a.u.), an APE of 17.5% in the velocity corresponds to an absolute deviation of approximately 8.7×10^{-3} a.u. For the electronic coefficient, an APE of 64% corresponds to a deviation of about 0.64 in $|c_0|$, meaning that the final electronic population differs substantially from the initial pure ground-state configuration.

The errors grow rapidly with increasing trajectory length whenever surface hops occur. For example, for the moderate-velocity case ($v = -0.05$ a.u.), the coordinate error increases from less than 1% at $N = 500$ to nearly 400% after 1000 steps, demonstrating a complete loss of reversibility. This behavior arises because, in the conventional velocity-Verlet implementation, the change of electronic state is only detected after completion of a nuclear time step. As a result, the potential energy surface governing the nuclear propagation during a hop differs depending on the direction of time, which breaks microscopic reversibility.

These results highlight that even relatively small inconsistencies in the treatment of surface hops can accumulate dramatically during longer trajectories. By contrast, when the piecewise propagation with line search is employed, the forward and backward trajectories retrace each other exactly within numerical precision, restoring strict time reversibility.

Table S1: Absolute percentage error (APE) between the initial conditions and the values obtained after one forward–backward propagation cycle using the naive velocity-Verlet implementation. The system is propagated forward for N integration steps and subsequently propagated backward for the same number of steps. The reported errors quantify the loss of reversibility introduced by the conventional integration scheme when surface hops occur. Results are shown for two initial velocities that trigger nonadiabatic transitions.

Observable	Initial velocity (a.u.)	$N = 100$	$N = 500$	$N = 1000$	$N = 1500$
q	-0.05	0.20	0.29	399	381
q	-0.5	0.053	0.19	0.29	400
v	-0.05	0.00037	0.037	4.32	17.48
v	-0.5	0.069	0.039	0.040	0.36
$\|c_0\|$	-0.05	0.928	5.70	25.86	64.49
$\|c_0\|$	-0.5	4.46	6.62	9.73	20.47

S10 The Uhlenbeck-Ornstein perturbation and detailed balance

The simple form of the TPS acceptance probability

$$P_{\mathrm{acc}}(X^{(o)} \rightarrow X^{(n)}) = h_A(\mathbf{\Gamma}_0^{(n)})\, h_B(\mathbf{\Gamma}_{L_n}^{(n)}) \min\left\{1, \frac{L_o}{L_n}\right\} \tag{S119}$$

presented in this work necessitates the use of a symmetric shooting frame modification scheme. Since only the velocity of the shooting frame is modified, we only need to show that the Uhlenbeck-Ornstein perturbation scheme,

$$v' = \alpha v + \sqrt{1-\alpha^2}\,\Delta v, \tag{S120}$$

where Δv is drawn from a Maxwell-Boltzmann velocity distribution, satisfies the detailed balance condition

$$\rho(v)P(v \rightarrow v') = \rho(v')P(v' \rightarrow v). \tag{S121}$$

The proof is as follows. Consider the rearrangement to express Δv in terms of v and v',

$$\Delta v = \frac{v' - \alpha v}{\sqrt{1-\alpha^2}}. \tag{S122}$$

The probability of drawing this specific Δv and thus proposing v' based on v is

$$P(v \rightarrow v') \propto \exp\left[-\frac{\beta m}{2}\left(\frac{v' - \alpha v}{\sqrt{1-\alpha^2}}\right)^2\right]. \tag{S123}$$

Combining with $\rho(v)$, this gives

$$\rho(v)P(v \rightarrow v') \propto \exp\left[-\frac{\beta m}{2}\left(v^2 + \left(\frac{v' - \alpha v}{\sqrt{1-\alpha^2}}\right)^2\right)\right]. \tag{S124}$$

The term inside the parenthesis of the exponent term reads

$$v^2 + \left(\frac{v' - \alpha v}{\sqrt{1 - \alpha^2}} \right)^2 = \frac{v^2 + v'^2 - 2\alpha v v'}{1 - \alpha^2}. \tag{S125}$$

Note here that this expression is symmetric with respect to the exchange of v and v', and so is the entire $\rho(v)P(v \to v')$. Therefore, there is

$$\frac{\rho(v)P(v \to v')}{\rho(v')P(v' \to v)} = 1, \tag{S126}$$

showing that this velocity modification scheme indeed preserves detailed balance.

S11 Analytical Properties of the Model System

The model system used in this work consists of two coupled harmonic diabatic potentials

$$\mathbf{H}(q) = \begin{bmatrix} \frac{\epsilon}{x_0^2}(q - x_0)^2 & V_c \\ V_c & \frac{\epsilon}{x_0^2}(q + x_0)^2 \end{bmatrix} . \tag{S127}$$

The specific values of x_0, ϵ, and V_c are given in the main text. Owing to the simple analytic form of the Hamiltonian, all quantities required for the dynamical simulations can be obtained analytically.

S11.1 Adiabatic Energies

Diagonalization of the diabatic Hamiltonian yields the adiabatic energies

$$E_{\pm}(q) = \left[q^2 + 1 \pm \sqrt{4q^2 + \left(\frac{V_c}{\epsilon}\right)^2} \right] \epsilon \ , \tag{S128}$$

where the symbols + and − denote the excited and ground electronic states, respectively. The two adiabatic surfaces form an avoided crossing centered at $q = 0$, where the energy gap between the states is minimal.

S11.2 Nonadiabatic Coupling

The nonadiabatic coupling between the two adiabatic states is defined as

$$d_{-+}(q) = \frac{\langle \psi_- \left| \frac{\mathrm{d}\mathbf{H}}{\mathrm{d}q} \right| \psi_+ \mid \rangle}{E_+ - E_-} \ . \tag{S129}$$

For the present model this quantity can be evaluated analytically, yielding

$$|d_{-+}(q)| = \frac{\epsilon |V_c|}{4\epsilon q^2 + V_c^2} . \tag{S130}$$

This expression shows that the nonadiabatic coupling is maximal at the avoided crossing ($q = 0$) and decays with increasing distance from the crossing seam.

S11.3 Dependence on the Electronic Coupling

The spatial localization of the nonadiabatic coupling depends strongly on the magnitude of the off-diagonal coupling parameter V_c. For small V_c, Eq. (S130) becomes sharply peaked near $q = 0$, leading to highly localized coupling between the two adiabatic states. In this regime, population transfer between the states occurs predominantly in the immediate vicinity of the crossing seam.

As V_c increases, the coupling becomes progressively broader in space. The rate of change of the coupling magnitude with respect to V_c is given by

$$\frac{\mathrm{d}|d_{-+}|}{\mathrm{d}V_c} = \frac{\epsilon(4\epsilon q^2 + V_c^2) - 2\epsilon V_c^2}{(4\epsilon q^2 + V_c^2)^2}. \tag{S131}$$

Because this dependence is approximately inverse quadratic in V_c, increasing the coupling parameter rapidly reduces the localization of the nonadiabatic coupling around the crossing seam. Consequently, electronic population transfer can occur over a wider range of nuclear coordinates.

S12 Proof of Rapid Equilibration

Even when choosing a much lower target temperature of 1000 K for the transition path sampling, the transition time of paths equilibrates rapidly to the baseline average at that temperature, as shown in Figure S1 from the initial 44,000 K. Rarely are longer paths sampled. This shows that by discarding the first 15% of all paths, we ensure that the path ensemble statistics are done under full thermal equilibrium.

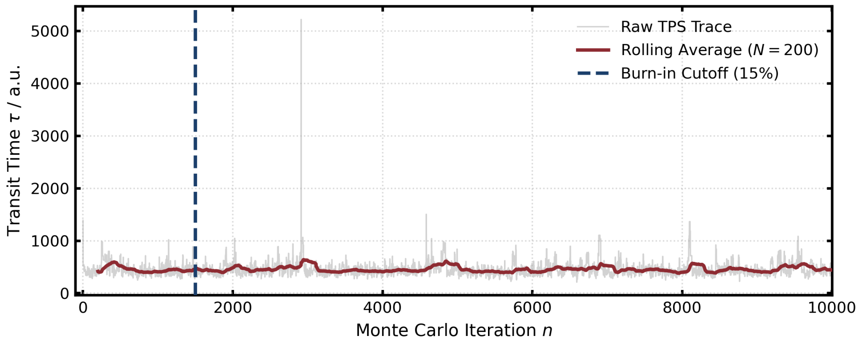

Figure S1: Path transition time with MC steps in the path space overlaying with the moving average of transition time of 200 paths at 1000 K. Under the Uhlenbeck-Ornstein perturbation $\alpha = 0.9$, equilibration is rapid (initial part of the gray line), and the discard ratio chosen is considered to be sufficient.

S13 The generation of lineage plot with TPS

This section provides an explanation to the production of 10 consecutive transition paths in Figure 2d in the main article. The readers are referred to the original figure for interpretations of legends and colors. Although the concept of position anchored time might sound confusing at first glance, it is actually a technique to formally tidy up the bundle of generated trajectories so that the phase-positional relationship between the shooting frame and the new path generated therefrom fits the intuitive understanding of the working process of TPS. Figure S2 shows the progression of the generation of transition paths with forward MC steps. This figure schematically shows the working of the TPS simulation by selecting a shooting point (green) and then generating a new trajectory from it. The anchored time axis is aligned so that the tip frame of the first generated trajectory that touches basin B is set to time 0. This ensures that the shooting point is visually the intersection of two consecutively generated trajectories.

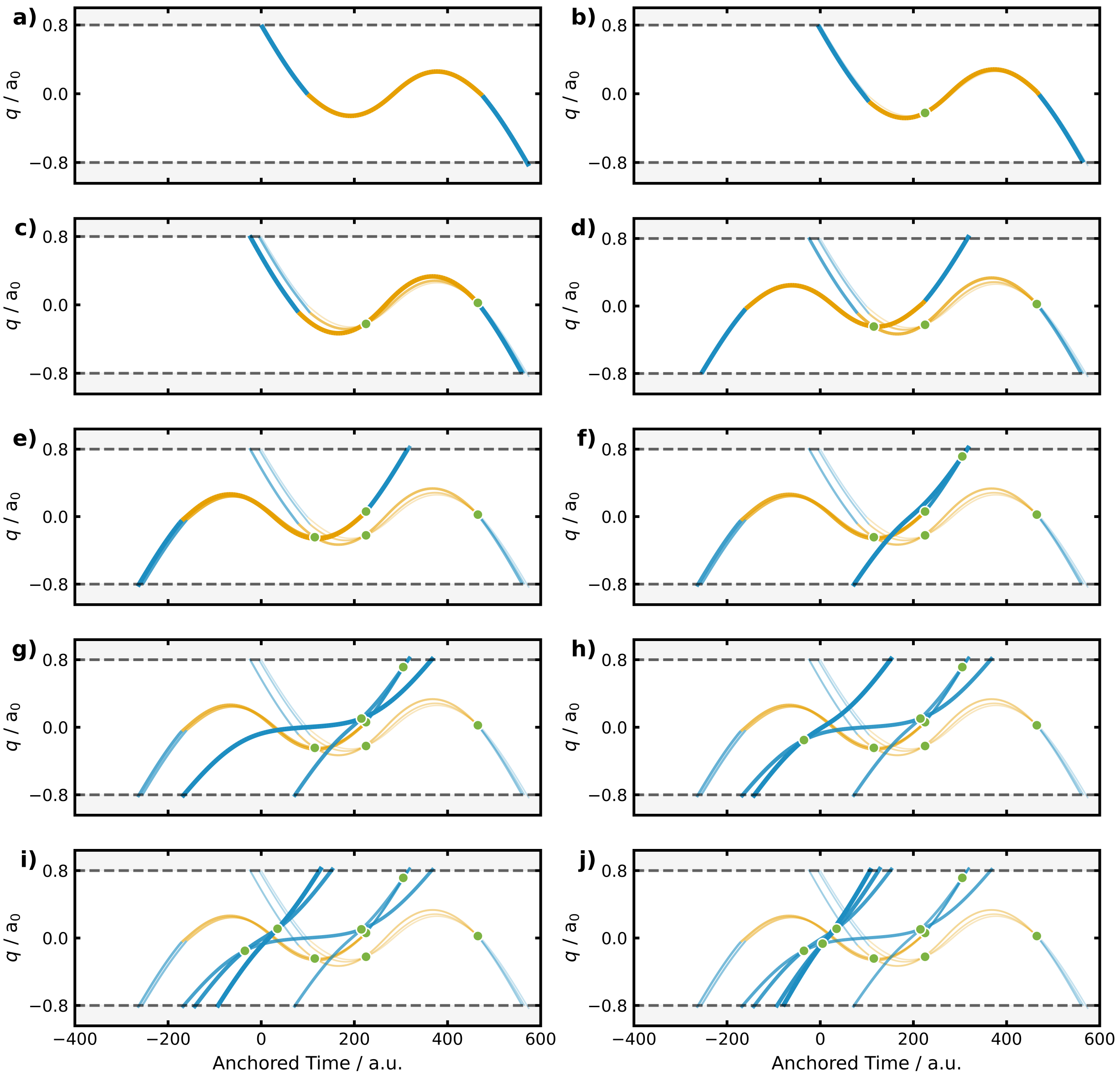

Figure S2: a)- i) The process of generating new transition paths with TPS. The anchored time axis is aligned so that the tip frame of the first generated trajectory that touches basin B is defined as time 0. Shooting frames are marked in green.

References

(S1) Amati, G.; Mannouch, J. R.; Richardson, J. O. Detailed balance in mixed quantum–classical mapping approaches. *The Journal of Chemical Physics* **2023**, *159*.

(S2) Gardiner, C. W. Handbook of Stochastic Methods for Physics, Chemistry and the Natural Sciences. *Springer Series in Synergetics* **1985**,

(S3) Mannouch, J. R.; Richardson, J. O. A mapping approach to surface hopping. *The Journal of Chemical Physics* **2023**, *158*.

(S4) Lawrence, J. E.; Mannouch, J. R.; Richardson, J. O. A size-consistent multi-state mapping approach to surface hopping. *The Journal of Chemical Physics* **2024**, *160*.

(S5) Plasser, F.; Granucci, G.; Pittner, J.; Barbatti, M.; Maurizio, P.; Hans, L. Surface hopping dynamics using a locally diabatic formalism: Charge transfer in the ethylene dimer cation and excited state dynamics in the 2-pyridone dimer. *The Journal of Chemical Physics* **2012**,
