# Supplementary material for: NATPS: Nonadiabatic Transition Path Sampling Using the Time-Reversible Mapping Approach to Surface Hopping
Source: J Phys Chem Lett. 2026 May 13;17(21):6014–23. doi: 10.1021/acs.jpclett.6c00910 (PMC13224161; doi:10.1021/acs.jpclett.6c00910)
Supplement: Supplementary file 2 [file jz6c00910_si_002.pdf]

jz-2026-00910u.R1

Name: Peer Review Information for "NATPS: Nonadiabatic Transition Path Sampling Using the Time-Reversible Mapping Approach to Surface Hopping"

First Round of Reviewer Comments

Reviewer: 1

Comments to the Author

The manuscript is devoted to the implementation of a method that allows the simulation of rare events in nonadiabatic molecular dynamics. In particular, the TPS approach is extended to consider nonadiabatic events by combining TPS with MASH. This is possible due to the favorable fetures of MASH (time reversibility and detailed balance). The algorithm is applied to a simple model system. Although limited to two states, the new method is interesting and potentially impactful. The manuscript is well written and certainly deserves to be published; I only have a few minor remarks.

1) he authors should provide a brief explanation of how the initial sampling trajectory was selected (top of page 12). Additionally, how were the shooting points generated?

2) In MASH, the starting values of the S vector is sampled randomly on the chosen hemisphere of the Block sphere (nothern or southern, according to the starting state). However, (judging from figure 2c) it does not seem that this is taken into

account in the present work. Why not?

3) In MASH, each trajectory has a weight proportional to |Sz|. However it seems to me that this is not considered in the present work. Maybe because |Sz| is always initialized to 1 (see the previous question)? The authors should clarify this point.

4) The mean transition time for nonadiabatic paths behaves erratically at high temperatures (figure 4b). Is this due to numerical instability or a distinct physical mechanism?

Reviewer: 2

Comments to the Author

In the manuscript entitled “NATPS: Nonadiabatic Transition Path Sampling Using the Time-Reversible Mapping Approach to Surface Hopping” by Xiran Yang et al., the authors present a new framework for nonadiabatic transition path sampling based on a time-reversible propagation scheme of the mapping approach to surface hopping. The method is applied to sample rare reactive trajectories in nonadiabatic dynamics efficiently. Since the simulation of rare nonadiabatic events remains an important challenge in nonadiabatic dynamics, I believe that this work could be of interest to the readership of The Journal of Physical Chemistry Letters. Here are my remarks.

Major points

1. While the present one-dimensional model appears sufficient for demonstrating the proof of concept of NATPS, the discussion of possible extension to multidimensional or multistate systems remains rather limited. Even without including an application to a

multidimensional or multistate system in the current study, the manuscript would benefit from a more detailed discussion of the challenges that may arise in such cases. For example, it would be helpful if the authors could comment on whether the procedure for locating the exact hopping time and performing piecewise continuous propagation remains straightforward in cases involving multiple hopping events or several electronic states. Such clarification would help readers better understand the scope and practical generality of the proposed framework.

2. Figure 3 and Table 1 support the conclusion that NATPS can obtain reactive trajectories using substantially fewer integration steps than brute-force MASH. However, because the efficiency ratio reported in Table 1 is defined in terms of the number of generated steps, it remains unclear whether NATPS is also more efficient in terms of the actual cost. In particular, the NATPS implementation involves additional procedures, such as shooting moves, forward and backward path propagation, and an iterative root search to locate hopping events, all of which may introduce non-negligible additional computational cost per step or per generated trajectory. Therefore, if readily available, providing a measure of the total computational cost that includes these additional procedures, for example in terms of actual CPU time, it would more clearly demonstrate the extent to which NATPS captures rare events more efficiently than brute-force MASH.

Minor points

3. Do well-known issues in surface hopping, such as overcoherence and the corresponding correction schemes, also remain relevant in the present NATPS framework?

4. Page 14, line 15. There appears to be a typo, as “and” seems to be repeated in the phrase “NATPS and and brute-force MASH.”

Reviewer: 3

Comments to the Author

This work addresses a key challenge in photochemistry: the simulation of rare nonadiabatic events. By combining MASH with TPS, the authors provide a rigorous framework with proofs of time-reversibility and detailed balance. The numerical demonstration is convincing and the method is promising. Nevertheless, several conceptual issues remain insufficiently clarified, and the following points should be carefully addressed before publication.

1. Scope of applicability

The introduction lists three mechanisms for rare nonadiabatic events: thermal activation, diffusion through complex landscapes, and escape from long-lived excited states. The model system focuses on barrier-limited and excited-state trapping, but diffusion-limited cases are not discussed. The authors should clarify whether NATPS is expected to work for diffusionlimited rare events.

More importantly, the manuscript does not explicitly state when NATPS should not be used. NATPS is not recommended for intrinsically fast, barrierless, strongly coupled processes where brute-force MASH already produces abundant reactive trajectories efficiently. Conversely, for barrier-limited reactions (e.g., low-temperature regime of the model), diffusion-limited encounters (if applicable), and excited-state trapping (e.g., weak coupling leading to long excited-state lifetimes), NATPS offers clear advantages. Linking each mechanism to concrete examples (as partly done for barrier and trapping) would help readers understand the precise applicability domain. These points need to be more clearly addressed. In addition, a few words should be added to emphasize what key information we may obtain by using NATPS in introduction. In introduction, we only feel that we may treat the slow nonadiabatic dynamics with NATPS by sampling path. However, what mechanism do we really know with this approach?

2. Equilibrium vs. non-equilibrium along a path

The authors prove detailed balance for MASH, which is an equilibrium concept. This raises a conceptual question that the manuscript does not explicitly address. My understanding is that in the path probability definition, only the initial point is drawn from the equilibrium distribution; the short-time transition probabilities satisfy detailed balance, but this does not imply that each intermediate configuration is in thermal equilibrium. On the contrary, the constraint that the trajectory must go from basin A to basin B forces it to pass through highenergy, low-probability regions (barrier tops, avoided crossings, excited states), which are far from equilibrium. Hence the path as a whole is non-equilibrium, and individual segments (except the start) are not required to be equilibrium samples. Detailed balance is

used to construct the path measure and ensure MCMC convergence, not to enforce equilibrium along the trajectory. Is this interpretation correct? The manuscript should clarify this point, as it is central to understanding the role of detailed balance in a non-equilibrium path ensemble. It is also important to really clarify the key issues connecting equilibrium and non-equilibrium side of this work.

3. Extensions beyond the current model

Can the NATPS framework be extended to more than two electronic states, to multi-dimensional nuclear degrees of freedom, and to QM/MM simulations? Please comment on any additional conditions or challenges.

4. Applicability to other mapping approaches

Other mapping-based nonadiabatic dynamics methods exist (Miller, Coker/Huo, Liu/He/Wu, Karpral, etc.). A careful discussion is needed regarding under what conditions these alternative methods could be substituted for MASH within the NATPS framework. Such a discussion would help readers assess the generality of the proposed path-sampling strategy.

5. Applicability to processes with different initial and final electronic states

Throughout the manuscript, the numerical demonstration is restricted to transitions where both basins A and B lie on the same electronic state (the ground state), with the excited state acting as an intermediate. Many nonadiabatic processes of interest, however, involve different electronic states for the reactant and product, e.g., photoinduced electron transfer energy transfer, or intersystem crossing. Can the NATPS framework be directly applied to such cases? If yes, what modifications (if any) are needed in the definition of A and B, the path probability, or the acceptance criteria? The authors should discuss this explicitly to clarify the scope of the method.

Reviewer: 4

Comments to the Author

This is a very interesting article about how to perform nonadiabatic transition path sampling given semiclassical trajectories. The ansatz of the authors is that because MASH

is time-reversible, one should be able to apply transition path sampling using MASH instead of newontina dynamics to generate trajectories.

Overall, the concept is very interesting and the paper can certainly be published. I wonder, though, if the article can be improved if the authors can make a few more things clear:

0. I wonder if perhaps the PIs can make a stronger case for why such a nonadiabatic scheme is required in the first place. After all, if we are looking for paths in nuclear trajectory space, will there ever be a time when the relevent trajectories do not resemble TPS path long the ground state?

Now admittedly, one might argue that I am looking for a path along an excited state, but then of course excited states have decay times… such that large barriers are likely not overcome before relaxation.

Bottom line: it would be good to have a slightly clearer view for why NA-TPS is necessary. After all, unless I am wrong, NA-TST-MASH is going to be quite expensive releative to adiabatic TPS.

1. I cannot tell from the algorithm as currently stated: can the PIs generate a reaction *rate* from TPS for these reactions? Is it accurate for spin-boson?

2. The authors talk about the canonical boltzmann distribution in eq 6, but is this distribution really relevant? After all, I thought for MASH one samples |S| = 3. Was this taken account of in the algorithm?

3. Also, for MASH, to recover reasonable results (especially correlation functions), as far as I am aware, one needs to weight the trajectories differently rather than weight each trajectory equivalently at all times. Am I missing something? Was this taken account of in the algorithm?

4. For Fig 3b, isn't the distribution in S (e.g. the phase distribution of S at the time of a hop) more interesting than the distribution in q?

Are all phases equally likely?

Small points: (a) What does the phrase "hyperparameters" connote on page 12?

(b) In Eq. 10, L0 and Ln are not defined.

(c) MASH can sometimes require a decoherence correction (which will break time reversbility)… at least, this is what I recall for Tully 3. I presume this point should be mentioned somewhere.

Author's Response to Peer Review Comments:

Dr.Johannes Dietschreit
University of Vienna
Institute of Theoretical Chemistry
Währinger Str. 17 A-1090
Vienna

johannes.dietschreit@univie.ac.at

Dear Editor,

We thank you for inviting us to this special issue and for forwarding the reviewers' comments. We are pleased that all four reviewers found the work interesting and potentially impactful, and we have carefully addressed each of their comments in the revised manuscript. Our replies are attached below.

We have expanded the discussion of the method's applicability domain, including its extension to multidimensional systems and multiple electronic states, and an explicit discussion of when NATPS is and is not expected to offer an advantage over direct simulation. We have further clarified that NATPS does not introduce any approximations beyond those of the underlying dynamics, and that the generated transition paths are correctly weighted by construction.

We believe the revised manuscript is significantly strengthened by these changes and hope that it is now suitable for publication in *The Journal of Physical Chemistry Letters*.

We look forward to hearing from you.

Sincerely,

Johannes Dietschreit
(on behalf of all authors)

Reply to reviewer #1

Comments to the Author

> The manuscript is devoted to the implementation of a method that allows the simulation of rare events in nonadiabatic molecular dynamics. In particular, the TPS approach is extended to consider nonadiabatic events by combining TPS with MASH. This is possible due to the favorable features of MASH (time reversibility and detailed balance). The algorithm is applied to a simple model system. Although limited to two states, the new method is interesting and potentially impactful. The manuscript is well written and certainly deserves to be published; I only have a few minor remarks.

We thank the reviewer for their positive evaluation of our work and their constructive feedback.

> The authors should provide a brief explanation of how the initial sampling trajectory was selected (top of page 12). Additionally, how were the shooting points generated?

We thank the reviewer for this question and have clarified the initialization and shooting moves in the text.

Changes to the manuscript:
The initial conditions for the trajectory from which the TPS sampling is started, were chosen as $v_{t=0} = -0.0087\ a_0/\mathrm{a.t.u.}$ (corresponding to a kinetic temperature of $44{,}000$ K) and $q_{t=0} = 1.0\ a_0$. Any alternative set of initial conditions that generated a trajectory connecting regions A and B would also have been acceptable. Shooting points are selected uniformly at random along a previously identified reactive trajectory. Then, only the velocities are perturbed, while the positions and electronic coefficients are retained from the preceding trajectory.

> In MASH, the starting values of the S vector is sampled randomly on the chosen hemisphere of the Block sphere (nothern or southern, according to the starting state). However, (judging from figure 2c) it does not seem that this is taken into account in the present work. Why not?

In standard MASH, each trajectory is initialized by sampling $\vec{S}$ randomly on the appropriate hemisphere. In the TPS framework employed here, however, we begin from a single reactive trajectory for which $\vec{S}$ is initialized at the south pole ($S_z = -1$). During

subsequent shooting moves, the position of $\vec{S}$ at the shooting point is inherited from the preceding accepted trajectory. As the path ensemble explores reactive trajectories through successive shooting moves, the influence of this specific initial choice of $\vec{S}$ is lost, since the decorrelation time of the spin vector is much shorter than the trajectory length. The generated path ensemble therefore correctly samples the reactive path distribution regardless of the initial $\vec{S}$ choice.

In the mentioned Figure 2c we only show an arbitrary reactive path to give the reader, who might be unfamiliar with MASH, a feeling for how hopping and S_z are connected. We have clarified the figure caption.

Changes to the manuscript:

b) An example of a reactive trajectory connecting basins A and B, with the color indicating the adiabatic PES the system evolves on. c) Corresponding time evolution of the spin-vector component S_z for the exemplary trajectory shown in panel b.

> In MASH, each trajectory has a weight proportional to |Sz|. However it seems to me that this is not considered in the present work. Maybe because |Sz| is always initialized to 1 (see the previous question)? The authors should clarify this point.

The different paths do not require additional weighting, as the employed shooting and perturbation scheme ensures that the generated transition paths follow the same distribution as the subset of reactive paths from the unperturbed path ensemble.

Changes to the manuscript:

This scheme ensures that the generated paths follow the same distribution as the subset of reactive paths from the equilibrium path distribution.

> The mean transition time for nonadiabatic paths behaves erratically at high temperatures (figure 4b). Is this due to numerical instability or a distinct physical mechanism?

Originally, we generated a fixed number of transition paths for all temperatures. As the distribution of transition times becomes broader with increasing temperature, the reliability of the estimate of the average transition time degrades with increasing temperature. This is not a prob-
lem of our method but a general statistical issue. To improve the estimate of $\langle\tau\rangle$, we have now

√

adapted the number of generated transition paths for each temperature such that

$\sigma_\tau(T)/ N_{\mathrm{TPS}}$ is approximately constant over the entire temperature range, where N_{TPS} is the number of generated transition paths, and $\sigma_\tau(T)$ is the standard deviation of transition times at temperature T. The additional sampling at high temperatures has removed the erratic behavior noticed by the reviewer.

Changes to the manuscript:

We notice that as temperature increases, the distribution of transition times broadens, and thus the standard deviation $\sigma_\tau(T)$ increases. To ensure reliable statistics from the path ensemble, we adapt the ensemble size N_{TPS} so as to keep the ratio $\frac{\sigma_\tau(T)}{\sqrt{N_{\mathrm{TPS}}}}$ approximately constant.

Addition to caption of Figure 4:

The size of the ensemble N_{TPS} increases with temperature such that the ratio between the $\sqrt{}$

standard deviation of transition times and $\overline{N_{TPS}}$ for a given temperature is constant.

Reply to reviewer #2

Comments to the Author

In the manuscript entitled “NATPS: Nonadiabatic Transition Path Sampling Using the Time-Reversible Mapping Approach to Surface Hopping” by Xiran Yang et al., the authors present a new framework for nonadiabatic transition path sampling based on a time-reversible propagation scheme of the mapping approach to surface hopping. The method is applied to sample rare reactive trajectories in nonadiabatic dynamics efficiently. Since the simulation of rare nonadiabatic events remains an important challenge in nonadiabatic dynamics, I believe that this work could be of interest to the readership of The Journal of Physical Chemistry Letters. Here are my remarks.

We sincerely thank the reviewer for their positive feedback and appreciate their productive comments. Below, we outline our point-by-point response to each comment and suggestion.

While the present one-dimensional model appears sufficient for demonstrating the proof of concept of NATPS, the discussion of possible extension to multidimensional or multistate systems remains rather limited. Even without including an application to a multidimensional or multistate system in the current study, the manuscript would benefit from a more detailed discussion of the challenges that may arise in such cases. For

example, it would be helpful if the authors could comment on whether the procedure for locating the exact hopping time and performing piecewise continuous propagation remains straightforward in cases involving multiple hopping events or several electronic states. Such clarification would help readers better understand the scope and practical generality of the proposed framework.

While MASH is derived for a two-level system, it can be extended to multistate systems using unSMASH or MISH. The search logic for the exact hopping time remains the same. However, multiple hops within a set time step is a known limitation of this time-reversible implementation, regardless of the number of states involved. More precisely, this constitutes a problem of any surface hopping method. The root problem is the discretization of time and cannot be resolved unless the simulation is genuinely continuous.

Regarding the dimensionality of the system, for the ease of analysis and presentation we selected a one-dimensional system, however, our method and code can directly be applied to any, multidimensional system that can be described by two electronic states. Such an application would then only face all the problems associated with the chosen molecular system and electronic structure method.

We thank the reviewer for raising this point and have added a more detailed discussion to the manuscript.

Changes to the manuscript:
In principle, any nonadiabatic dynamics method that supports time-reversible propagation and obeys detailed balance with respect to the equilibrium distribution can be combined with TPS to yield a nonadiabatic transition path sampling scheme. We selected MASH specifically because it rigorously satisfies both of these requirements. By building on a particular dynamics method, however, we naturally inherit both its strengths and its limitations. In its original formulation, MASH is strictly defined for two-level systems; extensions to multiple electronic states are possible via approximations such as unSMASH[1] or multi-state MASH, also termed MISH,[2] and combining these with NATPS is a natural direction for future work. While the present work uses a one-dimensional model for clarity of presentation, NATPS makes no assumptions about the number of nuclear degrees of freedom, and the implementation can be applied directly to multidimensional systems.

Figure 3 and Table 1 support the conclusion that NATPS can obtain reactive trajectories using substantially fewer integration steps than brute-force MASH. However, because the efficiency ratio reported in Table 1 is defined in

> terms of the number of generated steps, it remains unclear whether NATPS is also more efficient in terms of the actual cost. In particular, the NATPS implementation involves additional procedures, such as shooting moves, forward and backward path propagation, and an iterative root search to locate hopping events, all of which may introduce non-negligible additional computational cost per step or per generated trajectory. Therefore, if readily available, providing a measure of the total computational cost that includes these additional procedures, for example in terms of actual CPU time, it would more clearly demonstrate the extent to which NATPS captures rare events more efficiently than brute-force MASH.

We thank the reviewer for this thoughtful comment. The iterative root-search for locating the exact hopping time is applied in both the NATPS and the brute-force MASH simulations, so it contributes equally to the cost of both approaches and does not affect the efficiency ratio. Selecting a point along the current trajectory and perturbing the velocities are computationally negligible, as the cost of a single electronic structure evaluation (energies, forces, and couplings) vastly exceeds the overhead of the move itself in any realistic application. The forward and backward propagation from a shooting point produces trajectories of the same total length as a trajectory initiated from either basin boundary, so no additional steps are introduced. For these reasons, the number of integration steps remains the most meaningful and applicationindependent measure of computational cost, and we therefore prefer it over CPU timings obtained for an analytical one-dimensional model, which would not be representative of the real-world cost structure.

> Do well-known issues in surface hopping, such as overcoherence and the corresponding correction schemes, also remain relevant in the present NATPS framework?

We thank the reviewer for raising this point. Overcoherence is a well-known problem in fewestswitches surface hopping (FSSH), where the electronic wavefunction coefficients can remain in a superposition long after the system has committed to a single adiabatic surface. MASH largely avoids this issue by construction: because the active surface is determined deterministically from the spin vector, there is an inherent consistency between the electronic state and the nuclear trajectory that prevents the build-up of artificial coherence.[3] While quantum jumps can be applied to systematically improve MASH toward the full quantum-classical Liouville equation result, Richardson, Lawrence and Mannouch note that in most practical cases the bare MASH dynamics are already sufficiently accurate without any decoherence correction.[4] We therefore did not apply any such correction in the present work. Importantly, NATPS does not

introduce any coherence-related problems beyond those already present in the underlying MASH dynamics. As a path sampling framework, NATPS simply samples the trajectories that are produced by MASH with their correct probability; it does not alter the electronic equations of motion in any way.

Changes to the manuscript:
With respect to the electronic coefficients, we have to note that NATPS does not introduce any artifacts or approximations beyond those of the underlying MASH dynamics. Any correction scheme that can be applied to improve MASH, such as quantum jumps for decoherence, can in principle be incorporated into NATPS as well, though we find this unnecessary given the established accuracy of bare MASH for the systems considered here.[4]

> Page 14, line 15. There appears to be a typo, as “and” seems to be repeated in the phrase “NATPS and and brute-force MASH.”

We thank the reviewer for spotting this mistake. It has been fixed.

Reply to reviewer #3

Comments to the Author

This work addresses a key challenge in photochemistry: the simulation of rare nonadiabatic events. By combining MASH with TPS, the authors provide a rigorous framework with proofs of time-reversibility and detailed balance. The numerical demonstration is convincing and the method is promising. Nevertheless, several conceptual issues remain insufficiently clarified, and the following points should be carefully addressed before publication.

We sincerely thank the reviewer for their positive feedback and appreciate their productive comments. Below, we outline our point-by-point response to each comment and suggestion.

The introduction lists three mechanisms for rare nonadiabatic events: thermal activation, diffusion through complex landscapes, and escape from long-lived excited states. The model system focuses on barrier-limited and excited-state trapping, but diffusion-limited cases are not discussed. The authors should clarify whether NATPS is expected to work for diffusion-limited rare events.

We thank the reviewer for this question. TPS achieves its efficiency gain by eliminating the need to simulate the long waiting time between successive rare events. However, TPS does not shorten the transition paths themselves: if the reactive trajectories are intrinsically long, as is the case for diffusion-limited processes, where the system explores configuration space extensively before committing to the product state, each individual path is expensive to generate, and the efficiency advantage over brute-force simulation is reduced. This is a general limitation of TPS and is not specific to NATPS.

In the context of nonadiabatic photochemistry, the majority of rare events of interest are barrierlimited or involve trapping in a long-lived excited state, both of which produce short reactive trajectories and are therefore well-suited to TPS. Genuinely diffusion-limited nonadiabatic processes, such as solvent-controlled internal conversion where the geometry change required to reach the conical intersection is governed by viscous friction, are less common. NATPS would remain applicable in such cases, albeit with reduced efficiency compared to barrier-limited scenarios. We have added a brief clarification of this point to the manuscript.

Changes to the manuscript:
Regarding the efficiency of NATPS we have to note that, like TPS in general, it performs best when the reactive trajectories are short compared to the waiting time between

reactive events, as is the case for the studied model. For diffusion-limited nonadiabatic processes, where the transition paths themselves are long, the efficiency advantage is reduced, though the method remains applicable. However, for ultrafast processes, which are effectively barrierless, TPS does not provide any added benefit.

> The manuscript does not explicitly state when NATPS should not be used. NATPS is not recommended for intrinsically fast, barrierless, strongly coupled processes where brute-force MASH already produces abundant reactive trajectories efficiently. Conversely, for barrier-limited reactions (e.g., low-temperature regime of the model), diffusion-limited encounters (if applicable), and excited-state trapping (e.g., weak coupling leading to long excited-state lifetimes), NATPS offers clear advantages. Linking each mechanism to concrete examples (as partly done for barrier and trapping) would help readers understand the precise applicability domain. These points need to be more clearly addressed.

The applicability domain of NATPS is indeed an important point that deserves discussion. As noted in our response to the previous point, we have added a passage to the manuscript that addresses both concerns simultaneously.

Just as the reviewer noted, NATPS is most beneficial when reactive trajectories are short relative to the waiting time between reactive events. This covers the two most practically relevant scenarios in nonadiabatic photochemistry: barrier-limited reactions, where the system must overcome a free energy barrier to reach the region of strong nonadiabatic coupling, and excited-state trapping, where weak coupling leads to long-lived excited states and infrequent relaxation events. Both scenarios are illustrated by the low- and high-temperature regimes of our model system, respectively.

Conversely, NATPS offers no advantage for intrinsically fast, barrierless processes where bruteforce MASH already generates reactive trajectories efficiently, nor is it optimal for diffusionlimited processes where the transition paths themselves are long. These cases are now mentioned explicitly in the added text.

Changes to the manuscript:
Regarding the efficiency of NATPS we have to note that, like TPS in general, it performs best when the reactive trajectories are short compared to the waiting time between reactive events, as is the case for the studied model. For diffusion-limited nonadiabatic processes, where the transition paths themselves are long, the efficiency advantage is reduced, though the method remains applicable. However, for ultrafast processes, which are effectively barrierless, TPS does not provide any added benefit.

> A few words should be added to emphasize what key information we may obtain by using NATPS in introduction. In introduction, we only feel that we may treat the slow nonadiabatic dynamics with NATPS by sampling path. However, what mechanism do we really know with this approach?

We appreciate this question, as it addresses a very important point. NATPS in its pure form, as introduced in our work, can be used to sample reactive trajectories extremely effectively and efficiently for rare events in excited states (see the previous question for clarification of the areas of application) at a fraction of the brute-force computational cost. Having many such transition trajectories available, which is out of reach for rare nonadiabatic events for basically all computational methods, is essential for understanding the various reaction mechanisms that the process under investigation may exhibit. It helps us understand how reactions proceed, which reveals the underlying chemical mechanisms. We have clarified this point further in the introduction of the manuscript, as the reviewer suggested.

Changes to the manuscript:
To investigate these processes and understand the chemical reaction mechanisms involved, we need to study the reaction pathways of the relevant chemical reactions, and the presented method enables us to sample precisely these relevant paths in statistically sufficient quantities, something that has not been possible with previously available methods due to the enormous computational effort required. Furthermore, this makes it also possible to discover new chemical reaction mechanisms of nonadiabatic processes.

> The authors prove detailed balance for MASH, which is an equilibrium concept. This raises a conceptual question that the manuscript does not explicitly address. My understanding is that in the path probability definition, only the initial point is drawn from the equilibrium distribution; the short-time transition probabilities satisfy detailed balance, but this does not imply that each intermediate configuration is in thermal equilibrium. On the contrary, the constraint that the trajectory must go from basin A to basin B forces it to pass through highenergy, low-probability regions (barrier tops, avoided crossings, excited states), which are far from equilibrium. Hence the path as a whole is non-equilibrium, and individual segments (except the start) are not required to be equilibrium samples. Detailed balance is used to construct the path measure and ensure MCMC convergence, not to enforce equilibrium along the trajectory. Is this interpretation correct? The manuscript should clarify this point, as it is central to understanding the role of detailed balance in a non-equilibrium path ensemble. It is also important to really clarify the key issues connecting equilibrium and non-equilibrium side of this work.

We thank the reviewer for pointing out that we have to explain some key concepts of TPS more clearly. The reviewer is right that in TPS, the distribution of initial conditions is in equilibrium, and the underlying MASH dynamics obeys detailed balance, which is one of the features we use to prove the theoretical applicability of our method. This means that configurations are sampled according to the Boltzmann distribution. Forward and backward trajectories are statistically symmetric. TPS generates an equilibrium ensemble of trajectories in the sense that the probability distribution of transition paths is not altered by TPS compared to brute-force. In general terms, the system is therefore in equilibrium with respect to all possible trajectories. TPS, however, does not sample all possible, but rather reactive trajectories, *i.e.*, paths that go from basin A to basin B. Naturally, transition paths of chemical reactions show strong non-equilibrium behavior in the sense that a chemical reaction happens, however, the underlying dynamics satisfies detailed balance, *i.e.*, is in equilibrium. The sampling of transition paths is exactly what is intended with our and any other TPS method.

Lastly, we point out that (thermal) equilibrium is not a concept for single paths or single configurations. It only applies when taking into account the respective probability distributions.

Changes to the manuscript:
This scheme ensures that the generated paths follow the same distribution as the subset of reactive paths from the equilibrium path distribution. Hence, the probability distribution of transition paths is not altered by TPS compared to brute-force.

> Can the NATPS framework be extended to more than two electronic states, to multi-dimensional nuclear degrees of freedom, and to QM/MM simulations? Please comment on any additional conditions or challenges.

We thank the reviewer for this question.

Regarding multiple electronic states: in its original formulation, MASH is strictly defined for two-level systems. Extensions to more than two states are possible via approximations such as unSMASH[1] or multi-state MASH (MISH)[2], and combining these with NATPS is a natural direction for future work. Crucially, the TPS framework itself places no restriction on the number of electronic states; any multistate extension of MASH that retains time-reversibility and detailed balance can be straightforwardly combined with NATPS.

Regarding nuclear dimensionality: NATPS makes no assumptions about the number of nuclear degrees of freedom. The shooting moves, path acceptance criterion, and time-reversal operation are all formulated in terms of the full phase-space vector, regardless

of its dimension. The one-dimensional model was chosen purely for clarity of presentation and ease of analysis.

Regarding QM/MM: there is no conceptual obstacle to combining NATPS with a QM/MM description of the electronic structure. The only practical requirement is that the underlying dynamics method — here MASH — is implemented consistently with the QM/MM potential, which would apply to any trajectory-based nonadiabatic dynamics approach. The dominant computational cost in such a setting would be the electronic structure evaluation at each timestep, but as discussed in our response to Reviewer 2, point 2, the efficiency gain of NATPS over bruteforce MASH in terms of total integration steps directly translates to a corresponding reduction in the number of QM/MM evaluations required.

Changes to the manuscript:
In principle, any nonadiabatic dynamics method that supports time-reversible propagation and obeys detailed balance with respect to the equilibrium distribution can be combined with TPS to yield a nonadiabatic transition path sampling scheme.
In its original formulation, MASH is strictly defined for two-level systems; extensions to multiple electronic states are possible via approximations such as unSMASH[1] or multi-state MASH, also termed MISH,[2] and combining these with NATPS is a natural direction for future work. While the present work uses a one-dimensional model for clarity of presentation, NATPS makes no assumptions about the number of nuclear degrees of freedom, and the implementation can be applied directly to multidimensional systems.

> Other mapping-based nonadiabatic dynamics methods exist (Miller, Coker/Huo, Liu/He/Wu, Karpral, etc.). A careful discussion is needed regarding under what conditions these alternative methods could be substituted for MASH within the NATPS framework. Such a discussion would help readers assess the generality of the proposed path-sampling strategy.

We thank the reviewer for raising this point. The NATPS framework is not specific to MASH: any nonadiabatic dynamics method that supports time-reversible propagation and conserves the equilibrium distribution can in principle be combined with TPS in the same way. We selected MASH because it rigorously satisfies both of these requirements, as proven in our work.

We would like to emphasize that time-reversibility and conservation of the equilibrium distribution are not merely technical prerequisites for NATPS, they are fundamental properties that any physically consistent dynamics method should satisfy. Methods that violate these conditions, will produce incorrect dynamics regardless of whether they are

used within a path sampling framework. In some cases, such violations are accepted in exchange for practical advantages. Among the mapping-based methods cited by the reviewer, those that are time-reversible and conserve the correct equilibrium distribution could be substituted for MASH within NATPS without any modification to the path sampling framework itself.

We thank the reviewer for raising this point and have added a more detailed discussion to the manuscript.

Changes to the manuscript:
In principle, any nonadiabatic dynamics method that supports time-reversible propagation and obeys detailed balance with respect to the equilibrium distribution can be combined with TPS to yield a nonadiabatic transition path sampling scheme. We selected MASH specifically because it rigorously satisfies both of these requirements. By building on a particular dynamics method, however, we naturally inherit both its strengths and its limitations, as would we would for any other choice of dynamics method.

> Throughout the manuscript, the numerical demonstration is restricted to transitions where both basins A and B lie on the same electronic state (the ground state), with the excited state acting as an intermediate. Many nonadiabatic processes of interest, however, involve different electronic states for the reactant and product, e.g., photoinduced electron transfer energy transfer, or intersystem crossing. Can the NATPS framework be directly applied to such cases? If yes, what modifications (if any) are needed in the definition of A and B, the path probability, or the acceptance criteria? The authors should discuss this explicitly to clarify the scope of the method.

The NATPS framework places no restriction on how the stable states A and B are defined. In the present demonstration, both basins happen to lie on the ground electronic state, with the excited state acting as a kind of intermediate. In general, A and B can be defined in terms of any combination of nuclear and electronic degrees of freedom in the extended MASH phase space. For example, one could define B as a region of nuclear configuration space on the ground state and A as a region on an excited state, directly targeting processes such as photoinduced electron transfer, energy transfer, or intersystem crossing. No modifications to the path probability, acceptance criterion, or shooting procedure are required in such cases, as these are formulated entirely in terms of the extended phase space and are agnostic to the specific definition of the stable states.

We have clarified this point in the manuscript.

Changes to the manuscript:

It is important to note that A and B can be defined as regions of any extended phase space coordinate, or combinations thereof, including conditions on one or multiple electronic states. This allows NATPS to be directly applied to processes where the reactant and product reside on different electronic states without any modification to the method.

Reply to reviewer #4

Comments to the Author

> This is a very interesting article about how to perform nonadiabatic transition path sampling given semiclassical trajectories. The ansatz of the authors is that because MASH is time-reversible, one should be able to apply transition path sampling using MASH instead of newontina dynamics to generate trajectories. Overall, the concept is very interesting and the paper can certainly be published.

We sincerely thank the reviewer for their positive feedback and appreciate their productive comments. Below, we outline our point-by-point response to each comment and suggestion.

> I wonder if perhaps the PIs can make a stronger case for why such a nonadiabatic scheme is required in the first place. After all, if we are looking for paths in nuclear trajectory space, will there ever be a time when the relevent trajectories do not resemble TPS path along the ground state? Now admittedly, one might argue that I am looking for a path along an excited state, but then of course excited states have decay times… such that large barriers are likely not overcome before relaxation. Bottom line: it would be good to have a slightly clearer view for why NA-TPS is necessary. After all, unless I am wrong, NA-TST-MASH is going to be quite expensive relative to adiabatic TPS.

We appreciate this comment, as it gives us the opportunity to sharpen the motivation for NATPS in the manuscript.

The reviewer correctly identifies that many photoexcited systems undergo ultrafast internal conversion on timescales of tens to hundreds of femtoseconds, for which brute-force MASH is perfectly adequate and NATPS offers no advantage.

The most prominent examples where NATPS offers a significant advantage over brute-force dynamics are intersystem crossing (ISC) and reverse intersystem crossing (RISC). Singlet-triplet transitions are spin-forbidden, thus leading to pico to microseconds relaxation timescale, or even longer. Such processes are typically inaccessible for brute-force trajectory-based simulations. A second class of relevant processes involves systems where a barrier on the excited-state potential energy surface must be overcome before the system can reach the region of strong nonadiabatic coupling, such as conical intersections accessed via conformational changes in large flexible molecules. In both cases, the vast majority of computational effort in a brute-force

simulation is spent in regions far from the reactive event, and NATPS directly addresses this inefficiency.

We also wish to clarify that NATPS does not bias the dynamics in any way. The transition paths in the sampled ensemble are generated by the MASH equations of motion which conserve the Boltzmann distribution. The sampled reactive trajectories therefore represent the true mechanism of the rare event, without being artificially accelerated or modified.

Regarding the computational cost relative to adiabatic TPS: The path sampling algorithm itself is relatively insensitive to whether the associated dynamics is adiabatic or nonadiabatic. The computational cost of nonadiabatic dynamics is higher than simple ground state simulations due to the need for excited state gradients and interstate couplings. The relative cost of NATPS versus adiabatic TPS is therefore simply the relative cost of a MASH timestep versus a groundstate timestep, which is a property of the electronic structure method and not of the path sampling framework.

We have revised the introduction to make these points more explicit.

Changes to the manuscript:
Nonadiabatic processes are characterized by transitions between electronic states. They are central to modern photochemistry and molecular photophysics, encompassing a wide range of reactions like ring-opening, isomerization, and bond dissociation.[5,6] Although nonadiabatic transitions typically occur on femtosecond timescales,[7] the overall kinetics of excited-state processes may span many orders of magnitude. The apparent conflict between the contrasting timescales can be attributed to the low transition probabilities from weak electronic couplings and the low accessibility of electronically coupled regions, which may require thermally activated motion, diffusion through complex phase-space landscapes, or escape from long-lived excited-state minima.[7,8]

Consequently, many of the nonadiabatic processes manifest as kinetic rare events despite the ultrafast nature of the underlying microscopic dynamics. General examples include electron transfer[9], predissociation[10,11], photoisomerization[12–14], and intersystem crossing[15], which can span a wide range of timescales, from ultrafast to slow regimes. More concrete examples are the minor photodissociation channel of ammonia, $NH_3 \rightarrow NH + H_2$ with a quantum yield of less than one percent, requiring tens of thousands of trajectories to characterize statistically with brute-force surface hopping simulations,[16] and the intersystem crossing in sulfur-substituted nucleobases, which involves long-lived triplet states whose formation and decay are difficult to access computationally

due to the low probability of the relevant spin-forbidden nonadiabatic transitions.[17] These examples highlight the need to redirect the computational effort on the rare reactive events themselves, rather than the long waiting times between them, regardless of how such waiting times arise.

> I cannot tell from the algorithm as currently stated: can the PIs generate a reaction *rate* from TPS for these reactions? Is it accurate for spin-boson?

We thank the reviewer for this question. In the strict original sense of TPS, *i.e.*, Monte Carlo sampling in path space, a rate constant cannot be directly extracted from the generated transition path ensemble. This is because TPS samples only reactive trajectories connecting basins A and B, and therefore does not capture the waiting time in the stable states or recrossing trajectories, both of which are needed to recover a rate constant. This is sometimes referred to as the "lost clock" problem of TPS.

A well-established extension, transition interface sampling (TIS)[18,19], resolves this by combining the path ensemble with interface-crossing probabilities, enabling the calculation of rate constants. Combining NATPS with TIS is a natural next step and is highlighted as future work in the revised manuscript.

Regarding accuracy for the spin-boson model specifically: MASH has been benchmarked extensively on spin-boson systems and shown to recover Marcus theory rates accurately in the appropriate regime[20], which provides confidence that NATPS, which faithfully samples the MASH path ensemble, would inherit this accuracy. A direct benchmark of NATPS rate constants against spin-boson reference data is beyond the scope of the present work, but would be a natural validation target once the TIS extension is implemented.

Changes to the manuscript:
Future work will focus on the implementation in nonadiabatic dynamics packages such as SHARC[21,22] for applying NATPS to multidimensional molecular systems, and combining it with transition interface-based sampling (TIS) techniques[18,19] for the calculation of nonadiabatic rate constants.

> The authors talk about the canonical Boltzmann distribution in eq 6, but is this distribution really relevant? After all, I thought for MASH one samples |S| = 3. Was this taken account of in the algorithm?

We believe the reviewer may be thinking of Meyer–Miller–Stock–Thoss (MMST)-type mapping approaches, in which the classical spin vector is constrained to a sphere whose radius depends on the number of electronic states and the zero-point energy

parameter, and can exceed unity. In MASH, however, the spin vector lives strictly on the unit Bloch sphere ($|\mathbf{S}| = 1$), with $cos\theta$ sampled uniformly from $[-1,1]$. This is unambiguous in the original MASH formulation[3] and is exactly what is implemented in our work.

The canonical Boltzmann distribution in Eq. (6) therefore applies to the nuclear degrees of freedom in the standard way, combined with uniform sampling over the unit spin sphere, and no additional radius-related correction is required.

> For MASH, to recover reasonable results (especially correlation functions), as far as I am aware, one needs to weight the trajectories differently rather than weight each trajectory equivalently at all times. Am I missing something? Was this taken account of in the algorithm?

We thank the reviewer for raising this point, which connects to an important distinction between standard MASH dynamics and the NATPS framework.

In standard MASH, when computing time-correlation functions from an equilibrium ensemble of trajectories, the electronic observable estimators involve the Stratonovich-Weyl representation of the population operators, which introduces a factor of $2|S_z|$. However, this is a property of the *estimator* for electronic observables, not a trajectory weight in the sense of importance sampling.

In the NATPS framework, the situation is different in a fundamental way. Rather than computing correlation functions from an unbiased equilibrium ensemble, we are directly sampling the reactive path ensemble using a Metropolis-Hastings acceptance criterion. This ensures that the generated transition paths are distributed according to the correct reactive path probability by construction, without any need for additional reweighting. This is one of the key advantages of the TPS framework: the sampled paths are already correctly weighted, and the efficiency gain over brute-force simulation comes entirely from focusing computational effort on reactive trajectories rather than the long waiting times between them.

> For Fig 3b, isn't the distribution in S (e.g. the phase distribution of S at the time of a hop) more interesting than the distribution in q? Are all phases equally likely?

We thank the reviewer for the suggestion. We have replaced the histogram over hopping positions in Fig. 3b with a heat map showing both the hopping position distribution as well as the distribution of the x-y-plane component of the spin vector, in the form of the coherence angle

$\arctan\left(\frac{S_y}{S_x}\right)$. We find that while the hopping positions concentrate near the crossing seam, the angle ϕ is distributed uniformly. Hence, all phases are equally likely.

Changes to the manuscript: For the present one-dimensional model, this information is contained in the distribution of the nuclear geometries and electronic coefficients at the moment of hopping. Since at this time step $S_z = 0$, to express S_x and S_y compactly, we define

$$\phi = \arctan\left(\frac{S_y}{S_x}\right) \tag{R1}$$

as the angle of coherence. As shown in Fig. ??b, the distribution is centered symmetrically around the crossing seam located at $q = 0$, where the energy gap between the adiabatic states is minimal and the nonadiabatic coupling is the strongest. Since MASH only uses S_z to determine a hop, the distribution of ϕ is uniform. We note that $\tan x$ is undefined at $\pm\frac{\pi}{2}$, creating artificial nodes at $\pm\frac{\pi}{2}$ in Fig. ??.

Additionally, we have adapted Figure 3 and its caption.

> What does the phrase "hyperparameters" connote on page 12?

We have changed the diction to "parameters T and V_c", to avoid any confusion.

> In Eq. 10, L_0 and L_n are not defined.

We thank the reviewer for spotting this. We have defined the two symbols in the text.

> MASH can sometimes require a decoherence correction (which will break time reversbility)... at least, this is what I recall for Tully 3. I presume this point should be mentioned somewhere.

As the reviewer correctly notes, decoherence corrections are sometimes applied to MASH, and such corrections would in general break time-reversibility, which is a prerequisite for the TPS framework. However, as discussed by Richardson, Lawrence, and Mannouch,[4] bare MASH without any decoherence correction is already sufficiently accurate in most practical cases, precisely because the consistency between the spin vector and the active surface inherent to MASH largely prevents the build-up of artificial coherence that plagues FSSH. We therefore do not apply any decoherence correction in the present work. Should a decoherence correction be deemed necessary for a specific application, care would have to be taken to formulate it in a time-reversible manner before it could be incorporated into NATPS; the quantum-jump procedure of MASH[3]

offers in general a rigorous route to achieving this. We have added a note to the manuscript clarifying this point.

Changes to the manuscript:
With respect to the electronic coefficients, we have to note that NATPS does not introduce any artifacts or approximations beyond those of the underlying MASH dynamics. Any correction scheme that can be applied to improve MASH, such as quantum jumps for decoherence, can in principle be incorporated into NATPS as well, though we find this unnecessary given the established accuracy of bare MASH for the systems considered here.[4]

References

[1] Lawrence, J. E.; Mannouch, J. R.; Richardson, J. O. A size-consistent multi-state mapping approach to surface hopping. *The Journal of Chemical Physics* 2024, *160*.

[2] Runeson, J. E.; Manolopoulos, D. E. A multi-state mapping approach to surface hopping. *The Journal of Chemical Physics* 2023, *159*.

[3] Mannouch, J. R.; Richardson, J. O. A mapping approach to surface hopping. *The Journal of Chemical Physics* 2023, *158*.

[4] Richardson, J. O.; Lawrence, J. E.; Mannouch, J. R. Nonadiabatic Dynamics with the Mapping Approach to Surface Hopping (MASH). *Annual Review of Physical Chemistry* 2025, *76*, 663–687.

[5] Crespo-Hernández, C. E.; Cohen, B.; Hare, P. M.; Kohler, B. Ultrafast Excited-State Dynamics in Nucleic Acids. *Chemical Reviews* 2004, *104*, 1977–2020.

[6] Melchiorre, P. Introduction: Photochemical Catalytic Processes. *Chemical Reviews* 2022, *122*, 1483–1484.

[7] de Nalda, R., Bañares, L., Eds. *Ultrafast Phenomena in Molecular Sciences: Femtosecond Physics and Chemistry*; Springer Series in Chemical Physics; Springer International Publishing: Cham, Switzerland, 2014; Vol. 107; Proceedings or edited volume.

[8] Domcke, W., Yarkony, D. R., Köppel, H., Eds. *Conical Intersections: Theory, Computation and Experiment*; Advanced Series in Physical Chemistry; World Scientific Publishing Co.: Singapore, 2011; Vol. 17.

[9] Marcus, R. A. Chemical and Electrochemical Electron-Transfer Theory. *Annual Review of Physical Chemistry* 1964, *15*, 155–196.

[10] Kato, H.; Baba, M. Dynamics of Excited Molecules: Predissociation. *Chemical Reviews* 1995, *95*, 2311–2349.

[11] Sato, H. Photodissociation of Simple Molecules in the Gas Phase. *Chemical Reviews* 2001, *101*, 2687–2726.

[12] Güida, J.; Ramos, M.; Piro, O.; Aymonino, P. Infrared spectra of $K_2[RuCl_5NO]$ in two excited metastable states and the evidence for the NO linkage photoisomerization of metastable state I (MSI) in $[RuX_5 NO]^{2-}$ (X=Cl, CN). *Journal of Molecular Structure* 2002, *609*,
39–46.

[13] Ma, Y.-Z.; Premadasa, U. I.; Bryantsev, V. S.; Miles, A. R.; Ivanov, I. N.; Elgattar, A.; Liao, Y.; Doughty, B. Unravelling photoisomerization dynamics in a metastable-state photoacid. *Physical Chemistry Chemical Physics* 2024, *26*, 4062–4070.

[14] Ma, Y.; Premadasa, U. I.; Waters, M.; Kumar, N.; Doughty, B.; Laud, M.; Liao, Y.; Bryantsev, V. S. Accessing Transient Isomers in the Photoreaction of Metastable-State Photoacid. *ChemPhysChem* 2025, *26*.

[15] Penfold, T. J.; Gindensperger, E.; Daniel, C.; Marian, C. M. Spin-Vibronic Mechanism for Intersystem Crossing. *Chemical Reviews* 2018, *118*, 6975–7025.

[16] Bachmair, B.; Dietschreit, J. C. B.; González, L. Revisiting the intricate photodissociation mechanism of ammonia along the minor NH + H2 pathway. *Phys. Chem. Chem. Phys.* 2025, *27*, 8212–8220.

[17] Duwal, B.; Eder, I.; González, L.; Mai, S.; Ullrich, S. Tautomer aspects in the excited-state dynamics in 2-thiocytosine: intersystem crossing in the absence of the thiocarbonyl group. *Chem. Sci.* 2025, *16*, 15015–15028.

[18] van Erp, T. S.; Moroni, D.; Bolhuis, P. G. A novel path sampling method for the calculation of rate constants. *The Journal of Chemical Physics* 2003, *118*, 7762–7774.

[19] van Erp, T. S.; Bolhuis, P. G. Elaborating transition interface sampling methods. *Journal of Computational Physics* 2005, *205*, 157–181.

[20] Lawrence, J. E.; Mannouch, J. R.; Richardson, J. O. Recovering Marcus Theory Rates and Beyond without the Need for Decoherence Corrections: The Mapping Approach to Surface Hopping. *The Journal of Physical Chemistry Letters* 2024, *15*, 707–716, PMID: 38214476.

[21] Mai, S. et al. SHARC4.0: Surface Hopping Including Arbitrary Couplings — Program Package for Non-Adiabatic Dynamics. https://sharc-md.org/, 2025.

[22] Mai, S.; Marquetand, P.; González, L. Nonadiabatic dynamics: The SHARC approach. *Wiley Interdisciplinary Reviews: Computational Molecular Science* 2018, *8*, 1–23.

jz-2026-00910u.R2

Name: Peer Review Information for "NATPS: Nonadiabatic Transition Path Sampling Using the Time-Reversible Mapping Approach to Surface Hopping"

Second Round of Reviewer Comments

Reviewer: 2

Comments to the Author

I thank the authors for their detailed responses. The main concerns have been largely addressed, and I believe that the manuscript is now ready for the publication.

Reviewer: 3

Comments to the Author

I generally think that the authors address most of my questions. The only problem is that in their reply to my question on "The authors prove detailed balance for MASH, which is an equilibrium concept. This raises a conceptual question that the manuscript does not explicitly address. … Detailed balance is used to construct the

path measure and ensure MCMC convergence, not to enforce equilibrium along the

trajectory. Is this interpretation correct? The manuscript should clarify this point, as

it is central to understanding the role of detailed balance in a non-equilibrium path

ensemble. It is also important to really clarify the key issues connecting equilibrium

and non-equilibrium side of this work. ", their anwerrs seem to be fine, while the statements added in the manuscript seems to be very short and not clear. As this is a interesting discussion to link different research field, it should be more benefit to add more comprehensive/fundenmental discussions to make this work more reader-friendly.

Author's Response to Peer Review Comments:

Dr.Johannes Dietschreit
University of Vienna
Institute of Theoretical Chemistry
Währinger Str. 17 A-1090
Vienna
johannes.dietschreit@univie.ac.at

Dear Editor,

Thank you for handling the second round of review of our manuscript entitled "NATPS: Nonadiabatic Transition Path Sampling Using the Time-Reversible Mapping Approach to Surface Hopping". We are pleased that three of the four reviewers have approved the manuscript without further requests. In response to the remaining comment from Reviewer 3, we have extended the discussion of detailed balance in the manuscript as requested.

In addition, we have become aware that Jeremy Richardson has posted a manuscript on arXiv about transition path sampling with MASH since our original submission, which confirms the relevance of this topic. We have therefore added a sentence in the conclusion of our manuscript that refers to his work.

We hope that the manuscript is now ready for publication.

Sincerely,

Johannes Dietschreit
(on behalf of all authors)

Reply to reviewer #3

Original comment

> The authors prove detailed balance for MASH, which is an equilibrium concept. This raises a conceptual question that the manuscript does not explicitly address. My understanding is that in the path probability definition, only the initial point is drawn from the equilibrium distribution; the short-time transition probabilities satisfy detailed balance, but this does not imply that each intermediate configuration is in thermal equilibrium. On the contrary, the constraint that the trajectory must go from basin A to basin B forces it to pass through highenergy, low-probability regions (barrier tops, avoided crossings, excited states), which are far from equilibrium. Hence the path as a whole is non-equilibrium, and individual segments (except the start) are not required to be equilibrium samples. Detailed balance is used to construct the path measure and ensure MCMC convergence, not to enforce equilibrium along

> the trajectory. Is this interpretation correct? The manuscript should clarify this point, as it is central to understanding the role of detailed balance in a non-equilibrium path ensemble. It is also important to really clarify the key issues connecting equilibrium and non-equilibrium side of this work.

Original reply

We thank the reviewer for pointing out that we have to explain some key concepts of TPS more clearly. The reviewer is right that in TPS, the distribution of initial conditions is in equilibrium, and the underlying MASH dynamics obeys detailed balance, which is one of the features we use to prove the theoretical applicability of our method. This means that configurations are sampled according to the Boltzmann distribution. Forward and backward trajectories are statistically symmetric. TPS generates an equilibrium ensemble of trajectories in the sense that the probability distribution of transition paths is not altered by TPS compared to brute-force. In general terms, the system is therefore in equilibrium with respect to all possible trajectories. TPS, however, does not sample all possible, but rather reactive trajectories, *i.e.*, paths that go from basin A to basin B. Naturally, transition paths of chemical reactions show strong non-equilibrium behavior in the sense that a chemical reaction happens, however, the underlying dynamics satisfies detailed balance, *i.e.*, is in equilibrium. The sampling of transition paths is exactly what is intended with our and any other TPS method.

Lastly, we point out that (thermal) equilibrium is not a concept for single paths or single configurations. It only applies when taking into account the respective probability distributions.

Changes to the manuscript:
This scheme ensures that the generated paths follow the same distribution as the subset of reactive paths from the equilibrium path distribution. Hence, the probability distribution of transition paths is not altered by TPS compared to brute-force.

Reviewer's reply

> I generally think that the authors address most of my questions. The only problem is that in their reply to my question on "(See Original comment)". Their anwerrs seem to be fine, while the statements added in the manuscript seems to be very short and not clear. As this is a interesting discussion to link di!erent research field, it should be more benefit to add more comprehensive/fundenmental discussions to make this work more reader-friendly.

We appreciate the reviewer's feedback. We will add more to our current changes to the manuscript based on our previous replies.

Changes to the manuscript after Eq. (9):

The path probability defined in Eq. (8) corresponds to an equilibrium ensemble in path space. Since the underlying MASH dynamics obeys detailed balance and samples the Boltzmann distribution (Eq. (6)), a su"ciently long brute-force trajectory generates an ensemble of trajectory segments distributed according to $P[X(L)]$. Within this ensemble, transition paths are those trajectories that satisfy the

constraint $H_{AB}[X(L)] = 1$, *i.e.*, paths connecting basin A to basin B. Although such transition paths necessarily traverse high-energy and low-probability regions of configuration space and therefore exhibit non-equilibrium behavior at the level of individual trajectories, their statistical weight is defined with respect to the underlying equilibrium path ensemble. Equilibrium in this context thus refers to the probability distribution over paths, rather than to individual configurations along a trajectory.

Changes to the manuscript after Eq. (11):

A proof that this velocity perturbation scheme satisfies detailed balance is given in Section S10. As a result, the stationary distribution of the Markov chain coincides with the equilibrium path ensemble restricted to reactive trajectories. This means that TPS does not alter the probability distribution of transition paths compared to brute-force sampling, but provides a more e"cient means to sample them.
